# Computational Biophysical, Biochemical, and Evolutionary Signature of Human R-Spondin Family Proteins, the Member of Canonical Wnt/*β*-Catenin Signaling Pathway

**DOI:** 10.1155/2014/974316

**Published:** 2014-09-08

**Authors:** Ashish Ranjan Sharma, Chiranjib Chakraborty, Sang-Soo Lee, Garima Sharma, Jeong Kyo Yoon, C. George Priya Doss, Dong-Keun Song, Ju-Suk Nam

**Affiliations:** ^1^Institute for Skeletal Aging & Orthopedic Surgery, Hallym University-Chuncheon Sacred Heart Hospital, Chuncheon 200704, Republic of Korea; ^2^Institute for Skeletal Aging & Orthopedic Surgery, Hallym University Hospital, College of Medicine, Chuncheon-si, Gangwon-do 200-704, Republic of Korea; ^3^Department of Bioinformatics, School of Computer Sciences, Galgotias University, Greater Noida 203201, India; ^4^Center for Molecular Medicine, Maine Medial Center Research Institute, 81 Research Drive, Scarborough, ME 04074, USA; ^5^Medical Biotechnology Division, School of Biosciences and Technology, VIT University, Vellore, Tamil Nadu 632014, India

## Abstract

In human, Wnt/*β*-catenin signaling pathway plays a significant role in cell growth, cell development, and disease pathogenesis. Four human (Rspo)s are known to activate canonical Wnt/*β*-catenin signaling pathway. Presently, (Rspo)s serve as therapeutic target for several human diseases. Henceforth, basic understanding about the molecular properties of (Rspo)s is essential. We approached this issue by interpreting the biochemical and biophysical properties along with molecular evolution of (Rspo)s thorough computational algorithm methods. Our analysis shows that signal peptide length is roughly similar in (Rspo)s family along with similarity in aa distribution pattern. In Rspo3, four N-glycosylation sites were noted. All members are hydrophilic in nature and showed alike GRAVY values, approximately. Conversely, Rspo3 contains the maximum positively charged residues while Rspo4 includes the lowest. Four highly aligned blocks were recorded through Gblocks. Phylogenetic analysis shows Rspo4 is being rooted with Rspo2 and similarly Rspo3 and Rspo1 have the common point of origin. Through phylogenomics study, we developed a phylogenetic tree of sixty proteins (*n* = 60) with the orthologs and paralogs seed sequences. Protein-protein network was also illustrated. Results demonstrated in our study may help the future researchers to unfold significant physiological and therapeutic properties of (Rspo)s in various disease models.

## 1. Introduction

**Figure 1:**
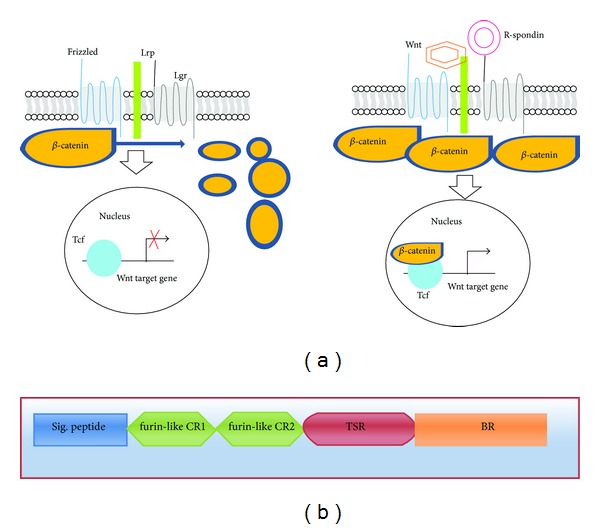
The role of (Rspo)s in canonical Wnt signaling pathway and the general architecture of (Rspo)s. (a) Schematic diagram of Wnt and R-spondin signaling models. In absence of Wnt ligand, constitutively synthesized cytoplasmic *β*-catenin is destroyed by the *β*-catenin destruction complex causing no *β*-catenin complex formation with T-cell transcription factor (Tcf)/Lef transcription factors for an active transcriptional response. Canonical Wnt signaling is instigated by the binding of Wnt ligands to the frizzled/LRP receptor complex which in turn deactivates the *β*-catenin destruction complex increasing its concentration in cytoplasm. The Wnt-frizzled/LRP complex-induced cytoplasmic buildup of *β*-catenin leads to its import into the nucleus and binding to (Tcf)/Lef transcription factors initiating transcription of Wnt targeted genes. (Rspo)s also act in similar manner but induce this unique property of enhancing Wnt activity by binding to recently discovered seven transmembrane G protein coupled receptors, Lgr (4, 5, and 6). (b) Schematic diagram shows the general domain architecture of human (Rspo)s. The architecture shows (i) signal peptide at N terminal end, (ii) two cysteine-rich furin-like repeats/domains, (iii) a single thrombospondin domain, and (iv) a basic amino-acid-rich domain at C-terminal basic region.

R-spondins (Rspo)s are a recently discovered family of genes that encodes cysteine-rich secretory proteins containing a thrombospondin type 1 domain/repeat-1 [1]. The (Rspo)s family includes four conserved proteins (Rspo1, Rspo2, Rspo3, and Rspo4), showing overall similarity of 40–60% sequence homology and domain organization [2]. Besides the existence of TSR-1 domain, all four (Rspo)s can be recognized by the existence of a carboxy-terminal region with positively charged amino acids and two furin-like cysteine-rich repeats adjacent to the amino terminus of the mature protein. Numerous studies have implicated (Rspo)s for acting synergistically with extracellular components of the Wnt signaling pathway (Figure 1) [3–5]. Studies showed close or overlapped gene expression of Wnt and (Rspo)s during developmental events, implying a possible coupling of the (Rspo)s with Wnt signaling [6–8]. Consistent with this, a significant reduction in mRNA expression of Rspo1 was observed in a Wnt1/3a double knockout mouse [1]. Rspo1 has been shown to augment Wnt signaling by interacting with the low-density lipoprotein receptor related protein 5 or 6 (LRP5/6) coreceptor and inhibiting Dickkopf-1 (Dkk-1) mediated receptor internalization [9]. Rspo2 deficient mice show death at early stages and have limb patterning defects associated with altered Wnt signaling [10, 11]. Rspo3 interacts with Frizzled 8 and LRP-6 and enhances Wnt ligand signaling [3, 4]. In addition to interaction with Wnt/*β*-catenin signaling, (Rspo)s can also regulate noncanonical Wnt signaling [12]. It was found that furin domain repeats are essential and sufficient for (Rspo)s to mediate Wnt-potentiating effects [13, 14]. Most recently, several studies conclusively determined that the (Rspo)s are the ligands for the leucine-rich repeat containing G protein-coupled receptor 4/5/6 (LGR4/5/6 receptors) [15–18].

Wnt signaling plays a fundamental role during fate determination steps of embryonic development and has been shown to govern process like cell differentiation, cell proliferation, and stem cell maintenance [19, 20]. Due to (Rspo)s ability to function as regulators of Wnt signaling pathways, various potential roles of (Rspo)s have been proposed and have been suggested as novel therapeutic targets [17, 21]. Rspo1 has been shown to control sex phenotypes between individuals. A study by Parma et al. [22] observed sex reversal due to the homozygous Rspo1 gene mutations in affected individuals. In addition, palmoplantar hyperkeratosis and predisposition to squamous cell carcinoma of the skin were also observed in these individuals. Rspo1 has also been recognized as a potent and specific mitogen for the gastrointestinal epithelium [13, 23]. Various studies have also implicated the importance of Rspo1 in skeletal biology. Rspo1 has been shown to synergize with Wnt3a to promote the process of osteoblast differentiation and inhibit the process of osteoclastogenesis by inducing expression of osteoprotegerin (OPG) [24–26]. Expression of Rspo2 has been shown to promote myogenesis via the Wnt/*β*-catenin signaling pathway in Xenopus [6]. A study with Rspo2 gene-targeted mutant mice observed that Rspo2 is requisite for normal development of several tissues, including craniofacial structures, lung, kidney, and limbs [27]. Moreover, study reported that Rspo2 is required for the maintenance of apical ectoderm ridge in the hind limbs of the mice. In other studies on Rspo2 mutant mice, hypoplasia and branching defects within the lungs were also being reported [11, 28]. It was observed that Lrp6-mediated Rspo2 signaling via the canonical Wnt pathway is essential for normal morphogenesis of the respiratory tract and for limbs as well [11]. Investigation into the genes responsible for coat features in domestic dogs revealed that Rspo2 is also supported in the Wnt-mediated hair follicle growth [29]. More recently, the role of recurrent Rspo2 gene fusion exclusively with APC mutations has been linked to the activation of Wnt signaling and colon tumorigenesis [30]. Like Rspo2 gene, recurrent Rspo3 gene fusions were also found to be associated with human colon tumors [30]. In recent time, it was proposed that Rspo3 gene may function along with Rspo2 gene in hind limb development, since the knockout of both Rspo2 and Rspo3 in limb mesenchymal cells caused more severe hind limb defects than those of Rspo2 mutant mice [31]. Rspo2 and Rspo3 genes were also identified for their oncogenic potential in mouse mammary tumor virus associated with mammary tumorigenesis in mice [32, 33]. Expression of Rspo4 has been shown to play a key role during nail development and mutations in Rspo4 gene results into absence of the nails in humans termed as anonychia/hyponychia congenita [34].

Given the diverse role of (Rspo)s in dynamic processes of life, like embryogenesis, tumor progression, angiogenesis, myogenesis, development of skeletal system, and so forth, we can expect (Rspo)s as vital therapeutic targets for a number of disabilities. Therefore, we tried to decipher biochemical, biophysical, molecular evolution, and protein-protein interaction characteristics of (Rspo)s by a series of computer based analysis. It may help us to understand the basic molecular properties of these molecules and thus their participation in critical events regulating essential life processes.

## 2. Materials and Methods

### 2.1. Data Mining for Human R-Spondin Protein Family Sequences and Their Feature of the Different Regions

We gathered the information on the sequences of human (Rspo)s family members based on searches in the National Centre for Biotechnology Information database (http://www.ncbi.nlm.nih.gov/protein) [35] and UniProt (http://www.uniprot.org/) [36, 37]. The FASTA formats of the sequences were further retrieved for analysis. To investigate the features of the primary structure such as the signal peptide in the protein chain and the chain other than the signal peptide portion, we used UniProt server (http://www.uniprot.org/), a database for information on proteins [36–38]. To understand signal peptide with “C-score” (predicted cleavage site value), “S-score” (the predicted signal peptide value), and “Y-score” (a combination of C- and S-scores), SignalP 4.0 server was used [39]. In addition, different repeats and domain in the R-spondin family members have been analysed using UniProt server.

### 2.2. Investigation of Amino Acid Distribution, Amino Acid Composition, and Some Parameters Related to the Primary Structure Such as Charge Distribution Analysis, Repetitive Structures, Cysteine Positions, and Disulphide Bonds of Human R-Spondin Family Proteins

To understand the amino acid distribution in the investigated proteins, we used protein calculator (http://spin.niddk.nih.gov/clore/Software/A205.html) [40]. In order to examine the amino acid prototype and protein sequence properties, such as amino acid composition percentage, high scoring hydrophobic segments, and tandem and periodic repeats of structure data of the human (Rspo)s, we used the statistical analysis of protein sequences (SAPS) [41], which is one of the most significant tools to bring out the details about protein sequence properties.

For the study of the secondary structural aspect of R-spondin family members such as cysteine positions and disulphide bond topology prediction, we used “SCRATCH protein predictor” for cysteine positions [42] as well as UniProt (http://www.uniprot.org/) [36, 37] server.

### 2.3. Structural Prediction of Thrombospondin-1 Domain Type 1 (TSP1) Repeats and Its Molecular Dynamics and Geometry

To understand the thrombospondin-1 domain type 1 repeats, we used the PDB file (1LSL.pdb) extracted from the protein data bank (http://www.rcsb.org); for further analysis see [43, 44]. The structure was visualized using Jmol Applet. We used InterPro, a database for protein families, domains, and functional sites, to understand domain structure [45]. The geometry of thrombospondin-1 domain type 1 repeats such as B factor plot, Omega plot, and FDS (fold deviation score) plot was developed using PDB server. Furthermore, we also developed Ramachandran plot for thrombospondin-1 domain type 1 repeats using PROCHECK server (http://www.ebi.ac.uk/thornton-srv/software/PROCHECK/).

### 2.4. Prediction of Glycosylation Sites

Analyses of the sequence location of the posttranslational modifications assist to determine the functional characteristics of the proteins. Glycosylation is a type of posttranslational modification (PTM) that assists in protein structural folding, transport, and different types of functions. We predicted the two kinds of glycosylation such as O-glycosylation and N-glycosylation sites by using the NetNGlyc and NetOGlyc servers of the four human (Rspo)s [46–48].

### 2.5. Prediction of R-Spondin Family Proteins Instability Index, Grand Average of Hydrophobicity (GRAVY), Aliphatic Index, and Total Number of Positively/Negatively Charged Residues

A comparison of the various biophysical and biochemical parameters of the proteins coded by the human (Rspo)s was carried out using the ProtParam tool from the ExPASy portal (http://web.expasy.org/protparam/) [49]. The different computed parameters for the (Rspo)s includes instability index, aliphatic index, grand average of hydrophobicity (GRAVY), total number of negative charged residues (Asp, Glu), and the total number of positive charged residues (Arg, Lys).

### 2.6. Prediction of Globularity in the R-Spondin Protein Family

Globular (globe-like) domain of the protein is having spherical domain. The ability to discover the functional sites of domains in proteins is becoming increasingly important. GlobPlot was used to predict the globularity in the domains [17]. The algorithm was as follows:

(1) $$\begin{array}{c} Ω\left(a_{i}\right)=\overset{i=1}{\underset{j=1}{\sum }}Ω\left(a_{j}\right)+\ln\left(i+1\right)\cdot P\left(a_{i}\right)\;\text{for}\;\;i=1,\ldots ,L. \end{array}$$

For the protein sequence which is used for analysis, the length of the sequence is *L*; Linding et al. [50] defined the sum function *Ω* as *P*(*a*_*i*_) ∈ *R*. *P*(*a*_*i*_) is the propensity of the *i*th amino acid and ln is the natural logarithm. The globularity in the domains of the regulatory subunit p85*α* was determined using the GlobPlot Web server.

### 2.7. Multiple Sequences Alignment (MSA) Analysis among R-Spondin Family Proteins

Four sequences of R-spondin family proteinswere used to understand the sequences similarity and alignment positions using MSA analysis. For that, we used clustal-omega to understand the sequence similarities and to elucidate the respective pairwise alignment scores. Clustal-omega has a graphical interface that is easy to use [51]. The clustal-omega server was organized on the basis of “progressive algorithm” [52] and the scoring system of the pairwise alignment algorithm is possibly the powerful component of the progressive algorithm. During the best alignment between *N* sequences, a computational complexity is found (*L* ^*N*^) for *N* sequences of length *L*. The basic algorithm to elucidate respective pairwise alignment scores is based on Needleman and Wunsch's algorithm [53].

Additionally, other MSA tools were used known as “multiple sequence comparison by log-expectation” (MUSCLE) to locate the conserved pattern across R-spondin protein family [54]. MUSCLE uses a function that can be described as the following log-expectation (LE) score function:

(2) $$\begin{array}{c} {\text{LE}}^{xy}=\left(1-f_{G}^{x}\right)\left(1-f_{G}^{y}\right)\log\underset{i}{\sum }\sum_{j}f_{i}^{x}\frac{f_{j}^{y}p_{ij}}{p_{i}p_{j}}. \end{array}$$

This function is a modified version of the log-average function expressed as follows:

(3) $$\begin{array}{c} {\text{LA}}^{xy}=\log\underset{i}{\sum }\sum_{j}f_{i}^{x}\frac{f_{j}^{y}p_{ij}}{p_{i}p_{j}}, \end{array}$$

where *i* and *j* are amino acid types; *p*_*i*_ is the background probability of *I*; *p*_*ij*_ is the joint probability of *i* and *j* being aligned to each other; *f*_*i*_^*x*^ is the observed frequency of *i* in column *x* of the first profile; and *f*_*G*_^*x*^ is the observed frequency of gaps in that column at position *x* in the family and likewise for position *y* in the second profile. The approximate probability *α*_*i*_^*x*^ of experimental amino acid *i* in location *x* can be derived from *fx*. The graphical yield of MUSCLE was visualised through JalView. Finally, Gblocks server was used to observe the aligned blocks of the sequences, which describes a set of conserved blocks from an MSA according to a set of simple requirements [55].

### 2.8. Multiple Sequences Alignment (MSA) Analysis of R-Spondin Family Proteins with Other Species

To understand the sequence similarity of human four (Rspo)s with other species, we used PhylomeDB server [56, 57]. This server performed homology searches by means of the Smith-Waterman algorithm [58] and ultimately filtered the sequences according to specific *e*-value and overlap cut-offs.

### 2.9. Analysis of Molecular Phylogenetics of Human R-Spondin Family Proteins

For the molecular phylogenetics, we used three servers to develop two phylogenetic trees. First we used accessible computer software and constructed the phylogenetic tree using Phylogeny.fr and performed computational biology [59]. This software uses several kinds of software for the workflow such as MUSCLE multiple alignment, Gblocks for the alignment curation, PhyML for the construction of the phylogenetic tree, and TreeDyn for the visualisation of phylogenetic tree. We have developed two types of the phylogenetic tree, namely, phylogram and cladogram (without branch distance). The phylogram depicted distances among protein sequences within the(Rspo)s. Then, another tree known as the “circular alpha phylogenetic tree” has been developed using MAFFT (version 7) [60]. Again using the four family sequences, we used clustal-omega to develop another phylogenetic tree [51]. The servers implemented either a neighbour-joining method or the bottom-up clustering method developed by Saitou and Nei [61] and the algorithm used a distance matrix to specify the distance between each pair of taxa. In this case, the matrix had a magnitude which is *N* × *N*. In this case, *N* is the number of points or nodes.

### 2.10. Prediction of Phylogenomics of Human R-Spondin Family Proteins Using Molecular Phylogenetics to Understand the Framework Topology of Other Related Species

To understand the phylogenomics of four human (Rspo)s and framework topology of other related species, we developed another phylogenetic tree using the sequence similarity of four human (Rspo)s with other species. For this analysis, we use PhylomeDB server, one of the largest phylogenetic repository [56, 57]. This server performed homology searches by means of the Smith-Waterman algorithm [58] and ultimately filtered the sequences according to specific *e*-value and overlap cut-offs. The server is a resulting collection of trees which characterize the full complement of evolutionary histories of all genes determined in a given genome. This has been entitled with the term phylome [59]. For phylogenomics analysis, the method used in this study is more closely a gene-centered method. And it is computationally more extensive compared to developing a family-based approach.

### 2.11. Understanding the Protein-Protein Interaction Network of R-Spondin Family Proteins

We have developed protein-protein interaction network using STRING server to understand the possible protein interactions with (Rspo)s [62, 63]. We developed four interaction networks, one for each (Rspo). Finally, we also developed scores to understand the interaction among possible interacting proteins with (Rspo)s.

## 3. Results 

### 3.1. Searched Data for Corresponding Proteins and Their Features Such as Signal Peptide, Repeats, and Domains

**Figure 2:**
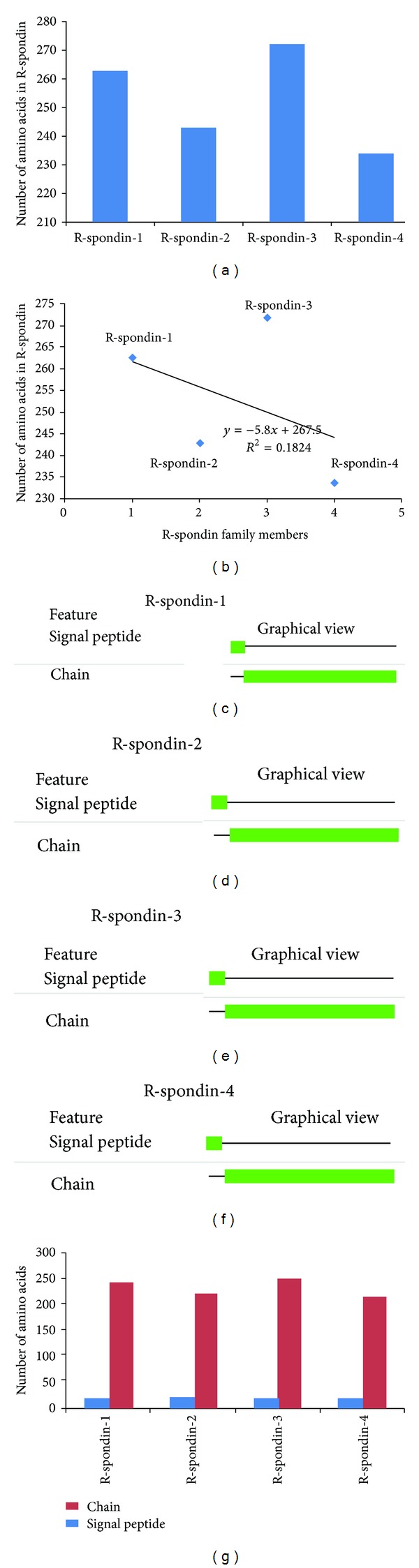
General architecture of human (Rspo)s in respect to amino acid sequence. (a) Comparison between the numbers of amino acids in all of the R-spondin family proteins. (b) Plot showing the scattered distribution of amino acid numbers along with the (Rspo)s and their correlations. ((c), (d), (e), (f)) Graphical overview of signal peptide and other parts of the amino acid chain-Rspo1, Rspo2, Rspo3, and Rspo4. (g) Comparison between number of amino acids in signal peptide and other parts of the protein in four human (Rspo)s.

**Figure 3:**
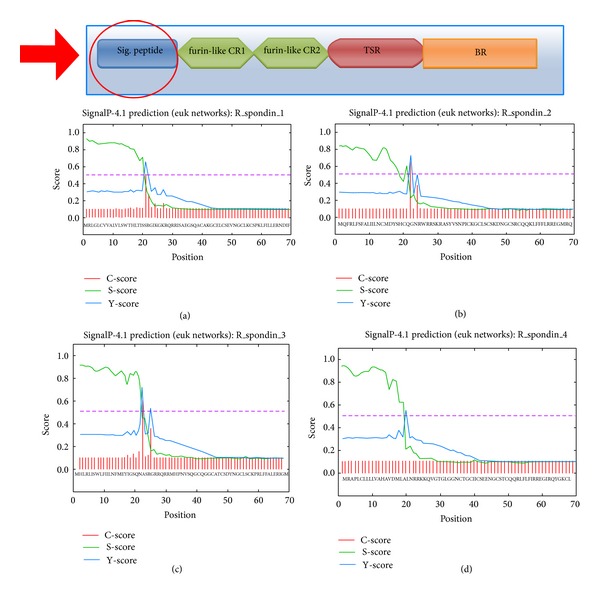
Predicted architecture of signal peptide of different human (Rspo)s with “C-score,” “S-score,” and “Y-score” (C-score represents predicted cleavage site value; S-score represents the predicted signal peptide value; Y-score represents a combination of C- and S-scores). (a) Rspo1, (b) Rspo2, (c) Rspo3, and (d) Rspo4 (the schematic diagram shows the location of signal peptide in the general domain architecture of human (Rspo)s and our region of analysis).

Supplementary Table S1 (available online at http://dx.doi.org/10.1155/2014/974316) shows the protein sequence information related to the human (Rspo)s analysed in this study, while the genes and proteins information related to human (Rspo)s have been displayed in Supplementary Table S2. The sequence lengths of Rspo1, Rspo2, Rspo3, and Rspo4 have been plotted in Figure 2(a). The figure shows that Rspo3 contains the highest sequence length of amino acids (aa) (272 aa), while Rspo4 contains the lowest sequence length (234 aa). Next, we plotted the sequence of amino acid number in the scattered distribution (*R* ^2^ = 0.1824) (Figure 2(b)). The features of the primary structure such as the signal peptide in the protein chain and the chain other than the signal peptide and information of different regions such as repeat and domain of human R-spondin family members were analysed. We depicted the position of regions, length, and graphical view of such regions in Figures 2(c), 2(d), 2(e), and 2(f). Thereafter, we compared the amino acid length of the signal peptide in the protein chains and the chain other than the signal peptide of these four proteins (Figure 2(g)). We observed that the length of the signal peptide portions is more or less similar (19 to 21 aa length) among the four proteins. Conversely, differences in the amino acid length have been noted in the chain other than the signal peptide portion where Rspo3 comprises the highest sequence length (251 aa) while Rspo4 contains the lowest sequence length (215 aa). Furthermore, we have analyzed the signal peptides of four human (Rspo)s and depicted their “C-score” (predicted cleavage site value), “S-score” (the predicted signal peptide value), and “Y-score” (a combination of C- and S-scores) (Figure 3).

### 3.2. Investigation of Amino Acid Distribution, Amino Acid Composition, and Some Parameters Related to the Primary Structure Such as Charge Distribution Analysis, Repetitive Structures, Cysteine Positions, and Disulphide Bonds of Human R-Spondin Protein Family

**Figure 4:**
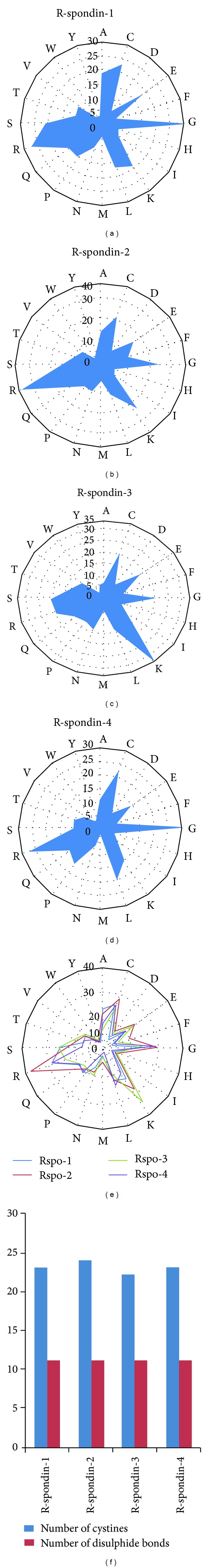
General amino acid distribution of amino acids in human (Rspo)s. (a) Rspo1, (b) Rspo2, (c) Rspo3, (d) Rspo4, (e) a general trend of amino acid distribution of amino acids for all human (Rspo)s where we have exposed the four protein's amino acid distribution at a time, and (f) comparison between number of cystine residue and disulphide bond in four human (Rspo)s.

Amino acid distributions of human R-spondin protein family have been reprinted in Figures 4(a), 4(b), 4(c), and 4(d). Furthermore, we exposed the four amino acid distributions at a time to understand the distribution pattern of these proteins (Figure 4(e)). The composition analysis of the amino acids of human (Rspo)s has been represented in Supplementary Table S3 and Supplementary Figure S1. From the calculated distribution of amino acids as well as the composition of the amino acids of human (Rspo)s, we found the following data: Rspo1 with highest Arg 27 (10.3%) and lowest Trp 3 (1.1%) and Tyr 3 (1.1%) both, Rspo2 with highest Arg 38 (11.1%) and lowest Trp 4 (1.6%), Rspo3 with highest Cys 22 (8.1%) and Ser 22 (8.1%) both and lowest Trp 3 (1.1%), and Rspo4 with highest Gly 27 (11.5%) and Arg 27 (11.5%) both and lowest Trp 3 (1.3%), respectively. From the distribution and composition of amino acid, it was noted that the highest of number of Arg residue was noted in the three proteins (Rspo1, Rspo2, and Rspo4), and the lowest number of residue was Trp in all of the R-spondin family proteins. The charge distribution analysis, repetitive structures, and cysteine positions of human (Rspo)s has been noted in Supplementary Table S3. Total numbers of cysteine and disulphide bonds present among (Rspo)s have been illustrated in Figure 4(f) showing maximum number of cysteine residues in Rspo2 protein (twenty-four). However, disulphide bonds are same in all of the human (Rspo)s (eleven).

### 3.3. Structural Prediction of Thrombospondin-1 Type 1 (TSP1) Repeats and Its Geometry

**Figure 5:**
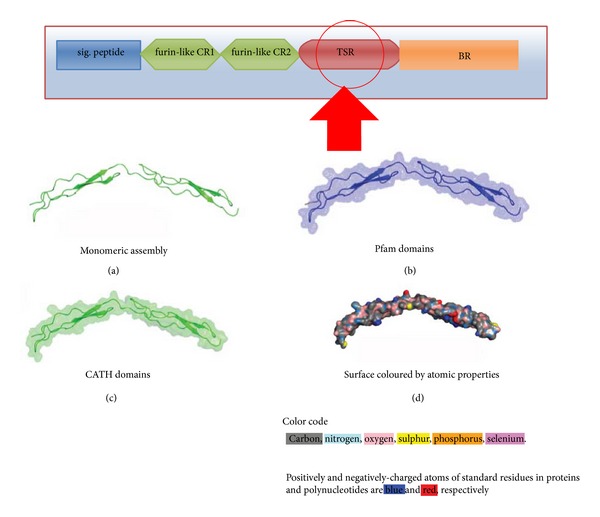
Unique backbone structure of thrombospondin-1 type 1 repeats/domain (TSR). (a) Monomeric assembly structure of TSR, (b) structure of TSR domain generated through Pfam domain database, (c) structure of TSR domain generated through CATH domain database, and (d) surface structure of TSR domain shows the atomic properties (the schematic diagram shows the location of thrombospondin-1 type 1 repeats/domain peptide in the general domain architecture of human (Rspo)s).

Human R-spondin family proteinscontain a thrombospondin type 1 domain type 1 repeats [1] (Figure 5(a)). The structure of monomeric assembly of the thrombospondin type 1 domain type 1 repeats has been depicted in Figure 5(b). This domain structure has been illustrated through the CATH and Pfam database and described in Figures 5(c) and 5(d). The surface structure of this domain has been developed with atomic properties described through different colours (Figure 5(e)). TSP1 domain(s) has been identified in a number of proteins, but generally in multiple copies. From this aspect, R-spondin is very unique since it has only one copy and predicted structure of this domain is hinge-like structure. This specific hinge-like structure of TSP1 domain may play a vital role in binding activity with the receptors. It has been found that TSR motifs especially the WSGWSSCSVSCG sequence are most significant for different neuronal responses such as neurite extension, neuronal survival, neuronal aggregation, and so forth [64].

B factors plot signifies the convolution of static and dynamic disorder in the crystal structure. While, dynamic disorder present in a crystal can be recognized through the local motions of individual atoms. Conversely, static disorder signifies the different atomic positions in a particular protein molecule [65]. Omega plot is helpful to understand the proper residue. Fold Deviation Score (FDS) plot is important to understand the structural geometry of the protein [32]. Ramachandran plot is also significant to comprehend residues in a generously allowed region [66]. Therefore, we developed the geometry of the thrombospondin type 1 domain type 1 repeats and the associated different geometry of these domain, such as B factor Plot, Omega plot, Fold Deviation Score (FDS) plot and Ramachandran plot and recorded in Supplementary Figures S2(a), S2(B), S2(C), and S2(D), respectively.

### 3.4. Prediction of Glycosylation Sites

**Figure 6:**
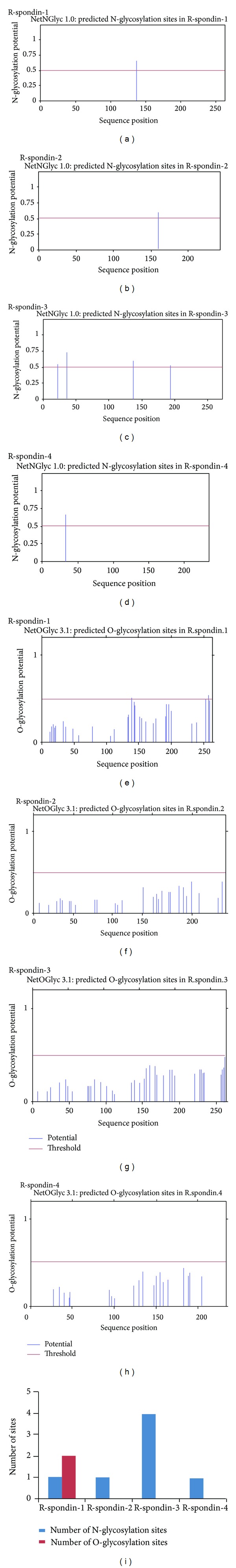
Predicted N-glycosylation and O-glycosylation potentialities and their positions in the different human (Rspo)s. (a) N-glycosylation potentialities of Rspo1, (b) N-glycosylation potentialities of Rspo2, (c) N-glycosylation potentialities of Rspo3, (d) N-glycosylation potentialities of Rspo4, (e) O-glycosylation potentialities of Rspo1, (f) O-glycosylation potentialities of Rspo2, (g) O-glycosylation potentialities of Rspo3, (h) O-glycosylation potentialities of Rspo4, and (i) comparison of predicted N-glycosylation and O-glycosylation sites for four human (Rspo)s.

Similar to phosphorylation, in some eukaryotic proteins, glycosylation plays a significant role in protein function and interaction during the signalling process [67]. In biophysical and biochemical point of view, N-glycosylation sites and O-glycosylation sites are important for functionality of the protein. In reviewing the presence of N-glycosylation sites (Supplementary Table S5) among (Rspo)s, we found the following: Rspo1 with 1 site (at the residue position of 137), Rspo2 with 1 site (at the residue position of 160), Rspo3 with 4 sites (at the residue position of 23, 36, 137 and 194) and Rspo4 with 1 site. The results of Rspo3 showed highest N-glycosylation sites. While reviewing the O-glycosylation potentiality and location (Supplementary Table S6), we found that only Rspo1 has one site. No other (Rspo)s have O-glycosylation sites. However, several O-glycosylation sites potentialities were recorded among (Rspo)s; although, the values of these sites were below the threshold limit (Figure 6).

### 3.5. Prediction of R-Spondin Family Proteins Instability Index, Grand Average of Hydrophobicity (GRAVY), Aliphatic Index, and Total Number of Positively/Negatively Charged Residues

**Figure 7:**
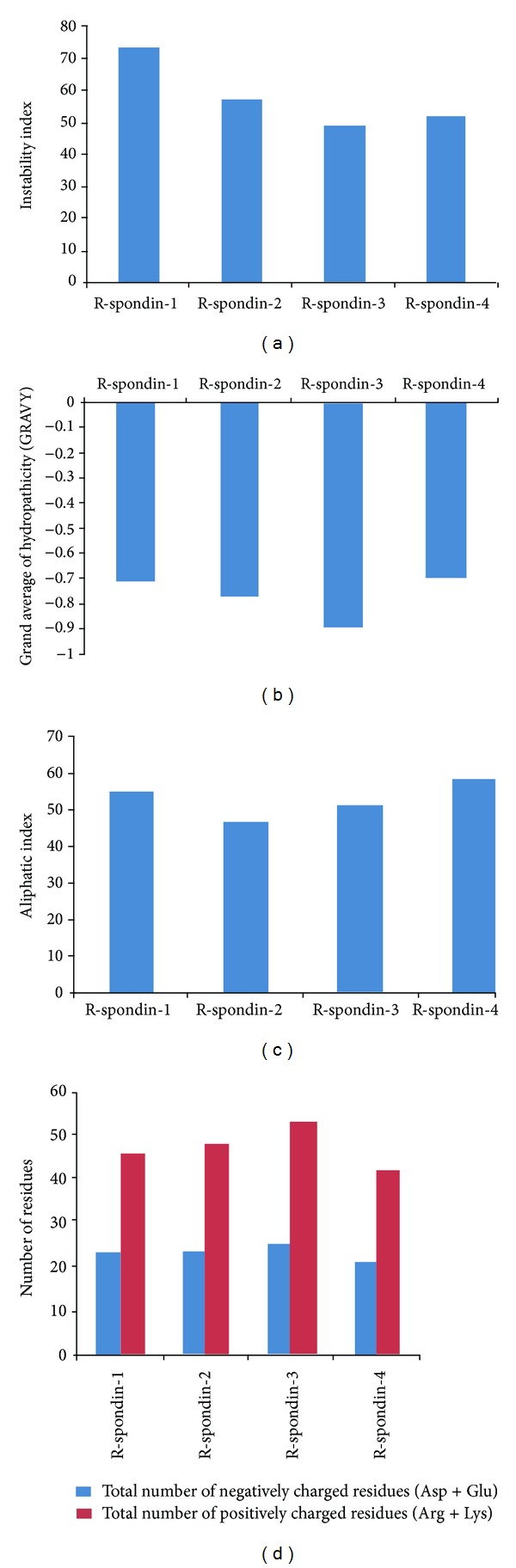
Comparison of biophysical and biochemical properties of four human (Rspo)s. (a) Comparison of instability index, (b) comparison of grand average of hydrophobicity (GRAVY), (c) comparison of aliphatic index, and (d) comparison of total number of positively/negatively charged residues.

The protein stability is associated with different structural properties and functionality of the proteins such as metabolic stability [68], protein-protein interactions [69], and so forth. An instability index provides the knowledge about a protein's stability, in particular in an* in vitro* environment. The instability index value greater than 40 designates an unstable protein, and one less than 40 designates a stable protein. Several factors such as the arrangement of amino acids in a sequence and some peptide bonds make* in vivo* proteins stable [70]. The results of our instability index analysis of the R-spondin family proteins are shown in Figure 7(a). The Rspo1 was found to have the highest instability index, whereas Rspo3 was found to have the lowest. Every R-spondin protein was found to be unstable as per their instability index, since the values are greater than 40. The changes in amino acid composition and hydrophobicity may have caused the observed distinct stability of the protein.

Kyte and Doolittle have formulated the scale of hydropathy in which the hydrophilic and hydrophobic possessions of amino acid chain are assessed in a protein [71]. Grand average of hydrophobicity (GRAVY) score can be computed as the sum of the hydropathy values for all the amino acids in a protein that can be divided by the total number of residues in the protein. Grand average of hydrophobicity (GRAVY) is associated with protein solubility. It has been noted that the positive GRAVY value is positively associated with hydrophobicity and negatively associated with the hydrophilicity. Because a more hydrophilic protein forms a larger amount of hydrogen bonds with water, therefore, the solubility is more. A ProtParam GRAVY study predicted grand average of hydrophobicity in the (Rspo)s (Figure 7(b)). Our analysis revealed that all (Rspo)s were hydrophilic in nature, Rspo3 being the most hydrophilic. The GRAVY value shows approximate similar values for the Rspo1, Rspo2, and Rspo4 (−0.717, −0.769, and −0.701, resp.).

The aliphatic index (AI) is very significant for understanding a protein, as it describes the relative volume occupied by aliphatic side chains such as alanine, valine, isoleucine and leucine. Aliphatic hydrophobicity is amplified with a rise in temperature and is, therefore, a positive factor enhancing the thermal stability of globular proteins [72]. Our analyses (Figure 7(c)) showed that, Rspo4 have the highest aliphatic index among (Rspo)s and the Rspo2 have the lowest. The AI value of Rspo1 (54.94) was approximately closer to the value of Rspo3 (51.58).

It has been reported that AI value is directly proportional to the structural stability of the protein. The procedure is generally used to calculate the AI of a protein [72, 73], which is as follows:

(4) $$\begin{array}{c} \text{AI}=X_{A}+aX_{V}+b\left(X_{I}+X_{L}\right), \end{array}$$

where, *X*_*A*_, *X*_*V*_, *X*_*I*_, and *X*_*L*_ represent the mole percentage of the four residues in a protein which are Ala, Val, Ile, and Leu, respectively. The notation “*a*” and “*b*” are coefficients representing the relative volumes of aliphatic side chains and the values are (*a* = 2.9 ± 0.1 and *b* = 3.9 ± 0.1), calculated from the volume occupied by the aliphatic amino acids in a protein.

Positively charged residues (PCR) and negatively charged residues (NCR) control several cell properties such as PCR controlled ribosomal velocity [74], NCR controlled K+ channels [75]. These two parameters are helpful to determine the topology of protein [76, 77]. A sum of Arg and Lys are calculated for the presence of the total number of positively charged residues in a protein. Conversely, totality of Asp and Glu are used to calculate the total number of negatively charged residues. Our analysis revealed that, Rspo3 contains the maximum number of positively charged residues while Rspo4 consisted of lowest number. Similarly, Rspo3 consisted of the highest number of negatively charged residues while Rspo4 had the lowest number (Figure 7(d)). The results signify that total numbers of positively charged residues are more than the total number of negatively charged residues for all (Rspo)s.

### 3.6. Prediction of Globularity in the R-Spondin Family Proteins

**Figure 8:**
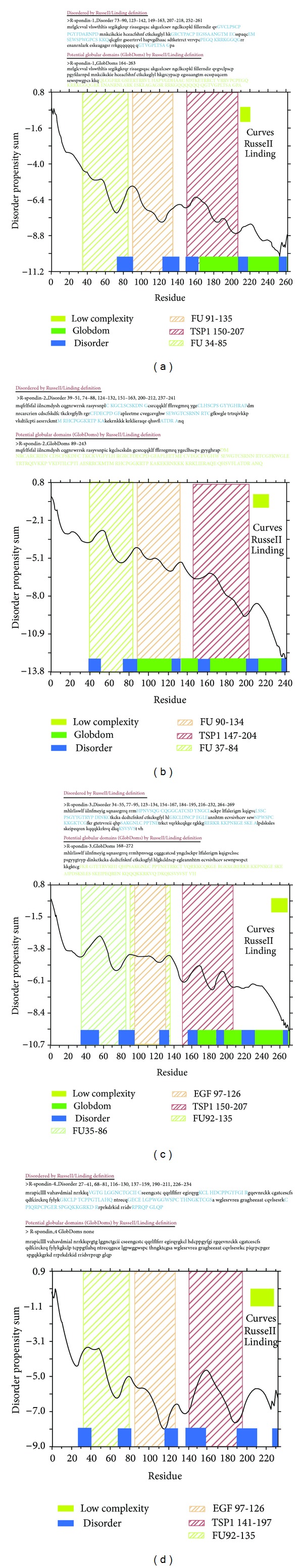
Globular domain gain/loss as a function of the variation between the four human (Rspo)s. The disorder propensity of the protein stretch was calculated using GlobPlot analyses to identify the disorder region (blue). The upper portion in the figure illustrates the differences between the amino acid sequence alignments among the domains. The tool uses a simple peak-finder algorithm to select the putative globular and disorder segments. (a) Rspo1, (b) Rspo2, (c) Rspo3, and (d) Rspo4.

From globular domains, several conventional concepts of protein science were initially developed and it challenge by essentially disordered domains [78]. It is frequently analysed to understand thestructure-function relationships, because the structure is having one or numerous catalytic or binding sites on its surface [79]. The globular domains which we analysed are shown in Figure 8. The amino acid sequence alignment in the upper portion of the figure illustrates the differences between the domains. All the proteins were found to contain disordered regions on its surfaces which are as following: Rspo1 (5), Rspo2 (6), Rspo3 (7) and Rspo4 (6). Although globular domain analysis found that Rspo1, Rspo2 and Rspo3 contains globular domain, but no globular domain was observed in Rspo4.

### 3.7. Multiple Sequences Alignment (MSA) Analysis among R-Spondin Protein Family

**Figure 9:**
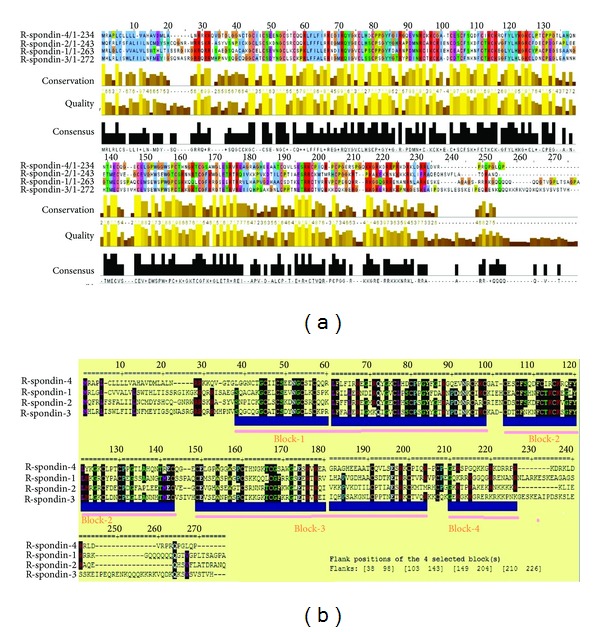
Multiple sequence alignment (MSA) of the different human (Rspo)s. (a) MSA output visualised through JalView and (b) the Gblocks results of human (Rspo)s show blocks from the alignments. The results show highly aligned four blocks.

The alignment of the (Rspo)s sequences using Clustal Omega is illustrated in supplementary Figure S3. The MUSCLE output was visualised through JalView and is shown in Figure 9(a). As mentioned, 37 small and large aligned divisions were found. We observed best aligned parts between the Rspo4 and Rspo2 sequences, as well as between Rspo4 and Rspo2. We also analysed the highly aligned blocks through Gblocks. The alignment results of Gblocks are shown in Figure 9(b) which shows four highly aligned blocks. From this result, we found highly conserved amino acids such as Leu, Arg, Ser, Gly, Cys, Asn, and Phe.

### 3.8. Multiple Sequences Alignment (MSA) Analysis of R-Spondin Family Proteins with Other Species

**Figure 10:**
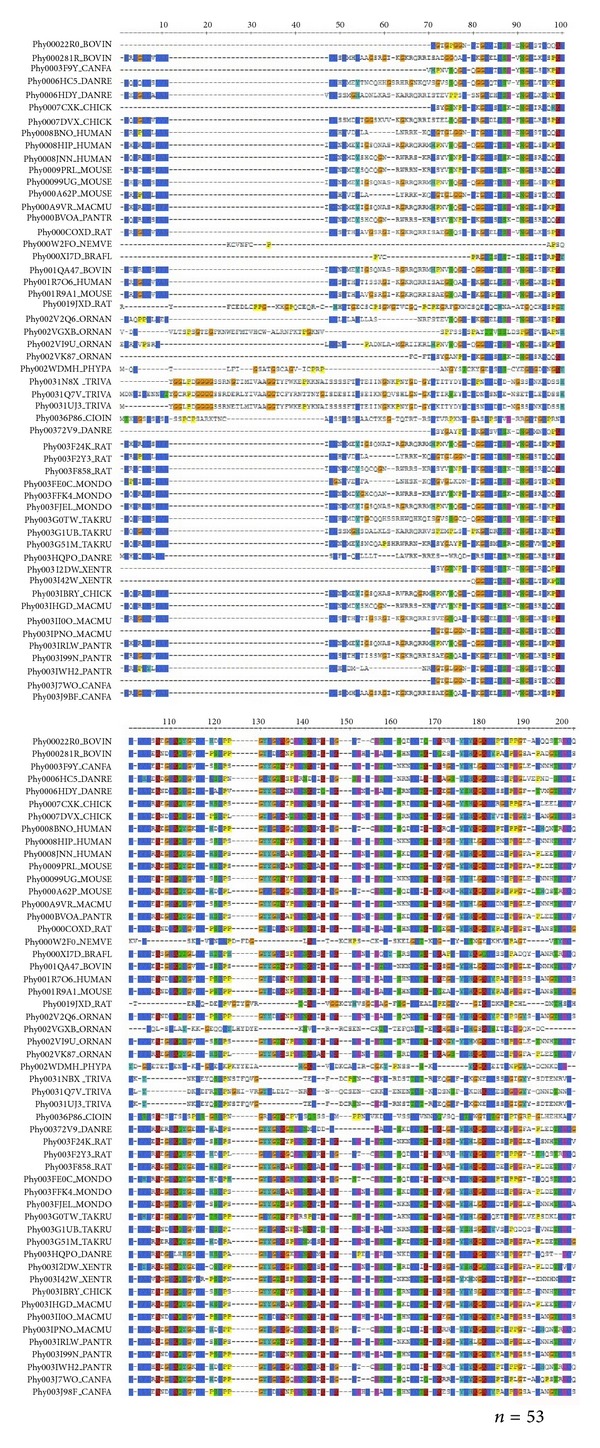
Multiple sequence alignment (MSA) of the different human (Rspo)s with other species (*n* = 53) which are having sequence similarity.

Thereafter, we performed MSA analysis of R-spondin family proteins with other species (*n* = 53). The MSA result is shown in Figure 10. The maximum conservation found was up to 270 sequence and some amino acids such as glycine, cysteine, valine, serine, proline, histidine, leucine and tyrosine were found highly conserved between the sequences.

### 3.9. Analysis of Molecular Phylogenetics of Human R-Spondin Family Proteins

**Figure 11:**
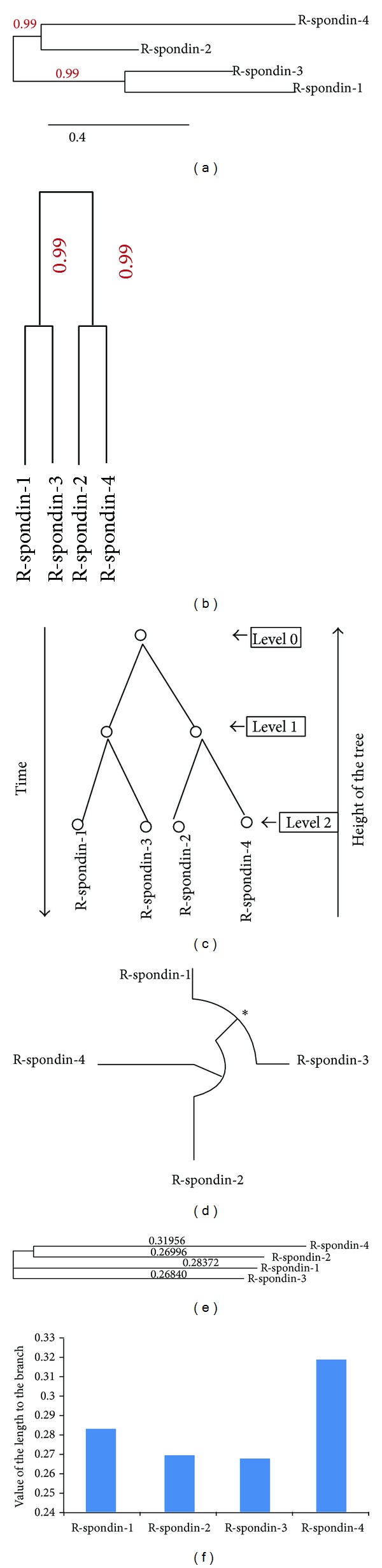
Phylogenetic analysis using different human (Rspo)s which shows the relationship between the family members. (a) A phylogenetic tree showing the evolution in the relationships between human (Rspo)s. Bootstrap values are pointed out at nodes. R-spondins protein family members names at the clade, (b) cladogram of protein sequences of the (Rspo)s members, (c) binary tree representation equivalent to cladogram, (d) phylogenetic tree (alpha circular type) reconstructed using the maximum likelihood method. The ∗ symbol adjacent to the node indicates the origin point of the proteins, (e) phylogenetic tree developed through clustal-omega server where the value of the branch length is mentioned immediately leading to the node, and (f) comparison between the value of the branch lengths.

Phylogram, cladogram and binary tree (equivalent to cladogram) have been depicted (Figures 11(a), 11(b), and 11(c)) and it demonstrates a significant relationship among the proteins of R-spondin family. A molecular phylogenetic analysis of R-spondin members would represent a significant feature of (Rspo)s evolution. In the constructed phylogenetic tree, the distance of branches was illustrated through the likelihood ratio mapping for evolutionary relationships among distinct members of R-spondin family. During the analysis of the tree algorithm, another figure have been described (Figure 11(c)) from the cladogram (Figure 11(b)), that clearly shows the phylogenetic tree rooted with ideal binary numbers (Figure 11(c)). The rooted tree contains two internal nodes and each internal node is further divided into two children nodes, highlighting proteins at their tips. We observed that the altitude of the binary tree was 2 stage. To cross check, other phylogenetic tree called “circular alpha phylogenetic tree”, was developed (Figure 11(d)) using MAFFT server. Developed tree resembled the first phylogenetic tree when compared (Figure 11(a)). Both the tree shows, Rspo4 being rooted with Rspo2 and likewise, Rspo3 and Rspo1 have the common point of origin. Again using the four sequences, we developed another phylogenetic tree using Clustal Omega (Figure 11(e)). This tree also showed that Rspo4 and Rspo2 have the common point of origin. We plotted the branch length from the tree in (Figure 11(f)), where Rspo4 showed longest branch length while Rspo3 had the shortest branch length.

### 3.10. Prediction of Phylogenomics of Human R-Spondin Proteins Using Molecular Phylogenetics to Understand the Framework Topology of Other Related Species

**Figure 12:**
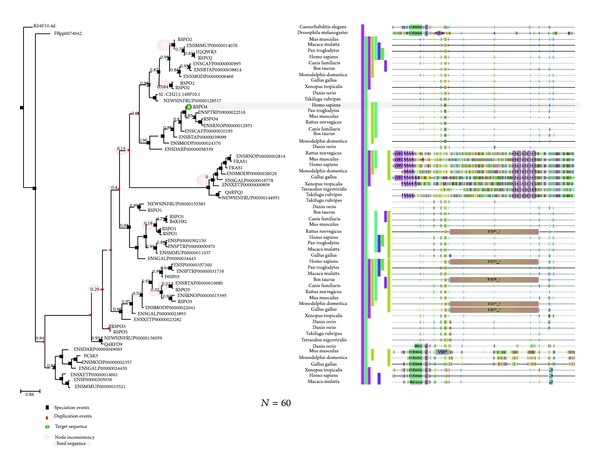
Phylogenomics of human four (Rspo)s and similar proteins from other species (*n* = 60) which are having sequence similarity. In front of the figure, the domain and sequence panel have been depicted which uses PFAM motifs, and the motifs are represented by different shapes.

Presently, the phylogenomics, the study of genomes from an evolutionary perspective, is one of the most significant branches to understand the molecular phylogenetics [80, 81]. Phylogenomics provides an understanding about the framework topology of other related species containing orthologous and paralogous genes. The phylogenomics and the framework topology may provide an understanding about the speciation event or duplication event [82, 83]. The phylogenomics (molecular phylogenetics) of human four (Rspo)s with other species have been depicted in Figure 12. Here, phylogenetic tree has been developed using sixty proteins (*n* = 60) and it is an interactive tree with the orthologs and paralogs of the seed sequences. Form Phylogenetic tree, it is very clear that R-spondin family is only distributed among vertebrate species. Our interactive tree shows the origin and evolution of R-spondin among vertebrate family members and it illustrated that non-vertebrate members (Drosophila and C. Elegans) are not having domains similar to that of R-spondins. We also specified the tree legend containing different color codes of the different tree nodes. In front of the figure, the domain and sequence panel have been illustrated showing PFAM motifs. The motifs are represented by different shapes. Inter-domain coding regions have been demonstrated as the standard amino acid colour codes and the gap regions are pointed up as a flat line.

Our tree not only describes the phylogenomics of the R-spondin family but also offers an ideal framework topology based on the biological knowledge of R-spondin family and other related sequences. Our result shows the state-of-the-art evolutionary patterns of R-spondin family and the related gene families.

### 3.11. Understanding the Protein-Protein Interaction Network of R-Spondin Protein Family

Complete knowledge about the protein-protein interaction networks offers direct and indirect interactions between proteins in a cell, helping us to depict a comprehensive description of cellular mechanisms and functions [84, 85].

**Figure 13:**
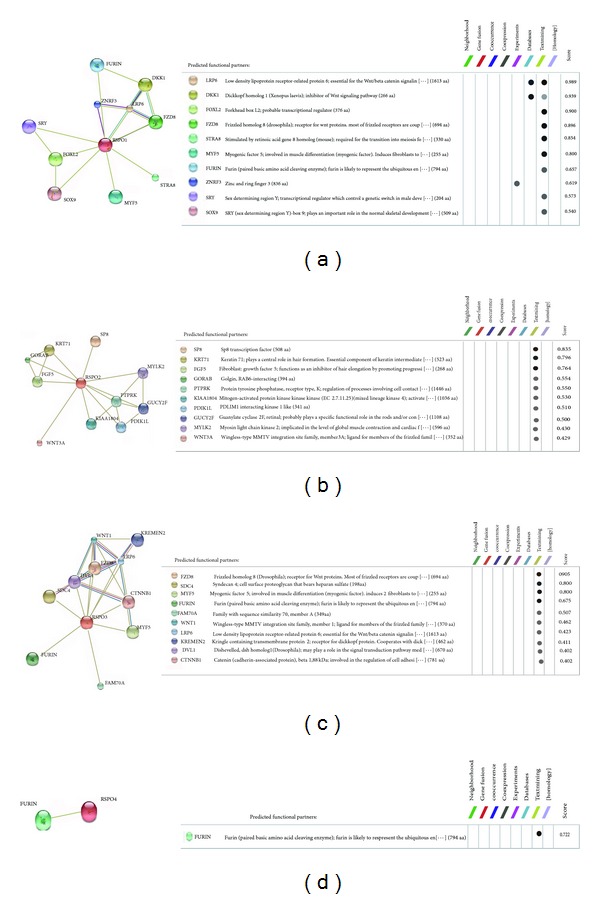
Protein-protein interaction network of R-spondin family proteins using STRING server. (a) Rspo1, (b) Rspo2, (c) Rspo3, and (d) Rspo4.

The protein-protein network of R-spondin protein family is illustrated in Figure 13. The input file for the development of protein-protein network of (Rspo)s has been is noted in the Supplementary Figure S4. Four different developed protein-protein interaction networks exemplified the different interactive proteins with the four members of R-spondin family. Rspo1 shows interaction network between FURIN, DKK1, ZNRF3, LRP6, FZR8, SRY, FOXL2, SOX9, MYF5, and STRA8 (Figure 13(a)). The interaction network is more condensed among DKK1, LRP6 and FZR8. Rspo2 shows interaction network between SP8, KRT71, FGF5, GORAB, PTPRK, KIAA1804, PDIK1L, GUCY2F, MYLK2, and WNT3A (Figure 13(b)). In this network, no condensed part was found. Rspo3 shows interaction network between FZD8, SDC4, MYF5, FURIN, FAM70A, WNT1, LRP6, KREMEN2, DVL1, and CTNNB1 (Figure 13(c)). The interaction network is more condensed among the proteins which are located in the upper portion of the network such as FZD8, SDC4, WNT1, LRP6, KREMEN2, DVL1, and CTNNB1. Rspo4 shows network between only one protein that is, FURIN (Figure 13(d)) and it is the shortest network among R-spondin protein family.

## 4. Discussion

R-spondin protein family is an immensely important protein family, which acts as a key regulator factor during vertebrate development and several signalling pathways, especially as agonists for the canonical Wnt/*β*-catenin signalling pathway [17]. Association with different diseases has been found with R-spondin family proteins. (Rspo)s are associated with various developmental stages as an essential regulator. For example, Rspo1 has been found to be associated with sex determination and skin differentiation [22]; Rspo2 is a crucial protein for development of limbs; lungs and hair follicles [11, 27, 86]; Rspo3 is essential for placental development [10] and Rspo4 is a significant protein for nail deployment [17]. (Rspo)s have therapeutic potential for various diseases such as skeletal diseases [87], inflammatory bowel disease and chemotherapy-induced mucositis [23], cancer [21], and diabetes [88]. Therefore, basic understanding about the biophysical, biochemical properties of (Rspo)s may provide more understanding about their functional mechanism associated with the diseases and the developmental processes. In this work, to decipher more about the biophysical, biochemical and evolutionary relationship of the R-spondin family, we carried out biophysical, biochemical, and evolutionary based computational mapping of human (Rspo)s.

In general, proteins have a small signal peptide sequence which helps them to enter into the secretory pathway. The N-terminal signal peptide sequence direct proteins to the membrane of the endoplasmic reticulum (ER) and initiate translocation into the ER lumen [89]. From our database analysis, we identified sequences similarity for signal peptides within (Rspo)s (20 to 21 sequence) (Figure 2) which corroborated to the finding of Kim et al. [2]. In addition to known findings, herein we analyzed signal peptides of human (Rspo)s in the more detail way along with their C-score, S-score and Y-score (Figure 3). Computational methods for estimating N-terminal signal peptides have been detected previously. But, our used server is an advanced tool which uses HMM-based better neural network scheme [39]. Using this tool, we have illustrated the predicted cleavage site value (C-score) in the signal peptide of human (Rspo)s where possible two signals are noted in a single signal peptidase cleavage site (Rspo2 and Rspo3) (Figure 3). Hiss and Schneider [89] revealed that long signal peptides mingle two or more signals of signal peptidase cleavage site.

From the amino acids distributed pattern of human (Rspo)s especially from the exposed distribution analysis at a time (Figure 4(e)), we observed the similarity of the amino acids distributed pattern is more or less same. However, Rspo1, Rspo2 and Rspo4 showed more similarity in the distribution pattern. At the same time, our analysis revealed identical amino acid composition pattern in the Rspo1, Rspo2 and Rspo4 (Figures 4(a), 4(b), 4(c) and 4(d)). Recently, it was reported that there is an association between amino acid composition and distribution with mutation. Researchers have shown the correlation between the amino acids distribution pattern; missense mutations and genetic disorders [90]. Conversely, amino acid composition was linked with the deleterious impact of mutations [91]. Therefore, amino acid composition and distributed pattern of human (Rspo)s may help to the future researcher to understand the impact and association with genetic disorders. Further analysis with Cys residues revealed that all these four (Rspo)s are Cys rich protein. Also, the Cys architecture and the disulphide bond pattern show a common architecture and may be necessary for the stability of these proteins (Figure 4(f)). Recent* in vitro* study with mass spectrometry documented the pattern of disulfide bonds between the 15 available Cys residues present in furin domains in (Rspo)s [14]. However, they found five free cysteine residues in Rspo2.

Our analysis found some glycosylation sites for (Rspo)s which may be necessary for their functionality and signalling process (Figure 6). Previously, Kamata et al. [1] has indicated the N-linked glycosylation sites for (Rspo)s. Our previous similar kind of computational analysis shows that the N-glycosylation sites and O-glycosylation sites are vital for the functionality of the proteins in the insulin signalling pathway proteins such as IRS and GLUT4 [67, 92]. However, identified O- and N-glycosylation sites by our analysis with (Rspo)s needs to be confirmed with molecular and biochemical experiments.

Previously, Kim et al. [2] and Nam et al. [4] performed multiple sequence analysis with four (Rspo)s. We also performed MSA among four (Rspo)s as well as with several other species proteins using different computational server (Figure 10). Compared to the previous analysis, our MSA investigation provides a very clear picture about the aligned and conserved residues with different colour codes visualised through JalView. We then analysed through Gblocks server to understand the conserve blocks within the R-spondin family. Our data showed four highly conserved blocks within depicted Gblocks (Figure 9). Furthermore, another MSA analysis of (Rspo)s with other species (*n* = 53) was performed to understand more conserved residues among different species where we found several small conserved blocks and residues such as glycine, cysteine, valine, serine, proline, histidine, leucine and tyrosine (Figure 10).

The evolutionary history of R-spondin family and the phylogenetic relationships prototype can be investigated through the molecular approach involving amino acid sequencing. Utilizing similar approach, we developed phylogenetic relationships among the members of the R-spondin family, and we found that Rspo4 and Rspo2 were siblings in 99% bootstrap replications and likewise, Rspo3 and Rspo1 were siblings in 99% bootstrap replications (Figure 11). Previously, de lau et al. [17] and our group also [4] analysed phylogenetic relationships. Here, we performed more advanced two types of phylogenetic analyses: (i) phylogenetic relationships pattern of R-spondin family (Figure 11) and (ii) phylogenetic relationships using R-spondin family using sixty species (*n* = 60) (Figure 12). Second one is the interactive tree with the orthologs and paralogs of the seed sequences which describe the phylogenomics of the R-spondin family and also determines evolutionary relationship of different species (Figure 12). This analysis directs the study towards next generation phylogenomics [93] which may be robust and alignment-free.

From our protein-protein interaction network analysis, we noted an interaction among the Rspo1 with the LRP6 and FZR8 receptor confirming them as candidate protein for Wnt signaling pathway (Figure 13(a)). Hao et al. [94] reported that LRP6 and FZD receptors are present on the membrane and these receptors permit the Wnt ligands to generate much stronger signals. The network of ZNRF3 with Rspo1 confirms that ZNRF3 is associated with Wnt receptor yield in an R-spondin sensitive manner [94]. The network also shows Rspo1 interaction with DKK1 (an antagonist of Wnt signaling). Binnerts et al. [9] reported that Rspo1 binds to the Kremen family of transmembrane proteins and it negatively regulates the LRP6 receptor through the DKK1-associated endocytosis. Due to the controlling property of individual's sex phenotype, Rspo1 networks with SRY and SOX9 protein [95]. The network of Rspo2 with FGF shows that damage Wnt signal directs to defective expression of the important apical ectodermal ridge maintenance factors, FGF4 and FGF8, which is related with the lung and limb development (Figure 13(b)). Similar to Rspo1, we observed a strong association between Rspo3 and the LRP6/FZR8 receptor as well as DVL for Wnt signaling pathway (Figure 13(c)). Rspo4 shows an interaction between FURIN proteins. It has been known that FURIN like domain is necessary for the activity of Rspo4. Blaydon et al. [96] demonstrated that mutations interrupting furin-like domains in Rspo4 may affect its signaling activity. Recent studies showed that (Rspo)s are the ligands for the leucine-rich repeat containing G protein-coupled receptor 4/5/6 (LGR4/5/6) receptors [15–18]. However, in our analysis we have not found any network between the (Rspo)s with LGR4/5/6. This might be due to the lack of updated data in server database (STRING database) containing information about the LGR4/5/6.

In summary, through computational analysis, we performed biophysical, biochemical, and evolutionary topology of human R-spondin family proteins. In this work, we have applied innovative and rapid approach to study the structural based biophysical, biochemical, and evolutionary relationship among (Rspo)s. The difficult and time-consuming nature of the experimental analysis led us to attempt to develop a cost-effective computational research of biophysical, biochemical and evolutionary topology of human R-spondin family. In this study, we have tried to highlight the possible potent sites for O- and N-glycosylation, distribution and conservation of amino acids and to predict phylogenetic and protein-protein interaction among (Rspo)s with the available data base. However, experimental biochemical and functional studies are required to further establish these finding. Our attempt to decipher the biophysical and biochemical properties of (Rspo)s may provide useful platform and a starting point for scientists to unfold significant physiological and therapeutic properties of R-spondin protein family in various disease models.

## Supplementary Material

Supplementary Table S1: Functional proteins associated (Rspo)s (Homo sapiens) and their protein IDs analyzed in this study.
Supplementary Table S2: (Rspo)s and their genes.
Supplementary Table S3: (Rspo)s and their compositional analysis, charge distribution analysis, repetitive structures, and cysteine positions.
Supplementary Table S4: Phosphorylation sites of (Rspo)s.
Supplementary Table S5: N-glycosylation sites of (Rspo)s.
Supplementary Table S6: O-glycosylation sites of (Rspo)s.
Supplementary Figure S1: Compositional analysis of (Rspo)s.
Supplementary Figure S2: Computational analysis of the geometry of the thrombospondin-1 domain type 1 (A) B factor plot, (B) Omega plot (C) FDS (fold deviation score) plot (D) Ramachandran plot.
Supplementary Figure S3: Sequence alignment (Rspo)s using Clustal Omega.
Supplementary Figure S4: Input file for protein–protein interaction analysis of (Rspo)s.
