# Supplementary material for: Computational Biophysical, Biochemical, and Evolutionary Signature of Human R-Spondin Family Proteins, the Member of Canonical Wnt/β-Catenin Signaling Pathway
Source: Biomed Res Int. 2014 Sep 8;2014:974316. doi: 10.1155/2014/974316 (PMC4172882; doi:10.1155/2014/974316)
Supplement: Supplementary file 1 — Supplementary Table S1: Functional proteins associated (Rspo)s (Homo sapiens) and their protein IDs analyzed in this study. Supplementary Table S2: (Rspo)s and their genes. Supplementary Table S3: (Rspo)s and their compositional analysis, charge distribution analysis, repetitive structures, and cysteine positions. Supplementary Table S4: Phosphorylation sites of (Rspo)s. Supplementary Table S5: N-glycosylation sites of (Rspo)s. Supplementary Table S6: O-glycosylation sites of (Rspo)s. Supplementary Figure S1: Compositional analysis of (Rspo)s. Supplementary Figure S2: Computational analysis of the geometry of the thrombospondin-1 domain type 1 (A) B factor plot, (B) Omega plot (C) FDS (fold deviation score) plot (D) Ramachandran plot. Supplementary Figure S3: Sequence alignment (Rspo)s using Clustal Omega. Supplementary Figure S4: Input file for protein–protein interaction analysis of (Rspo)s. [file 974316.f1.pdf]

Supplementary Table 1

Table S1 Functional proteins associated R-spondin (Homo sapiens) and their protein IDs analyzed in this study.

Seq No.	Protein	Protein/ Gene ID	Protein length	Other Information
1	R-spondin-1	NP_001033722.1	263 aa	DEFINITION: R-spondin-1 isoform 1 precursor [Homo sapiens].<br>ACCESSION: NP_001033722<br>VERSION: NP_001033722.1 GI:84490388
2	R-spondin-2	NP_848660.3	243 aa	DEFINITION: R-spondin-2 isoform 1 precursor [Homo sapiens].<br>ACCESSION: NP_848660<br>VERSION : NP_848660.3 GI:222446611
3	R-spondin-3	NP_116173.2	272 aa	DEFINITION R-spondin-3 precursor [Homo sapiens].<br>ACCESSION NP_116173<br>VERSION NP_116173.2 GI:18490982
4	R-spondin-4	NP_001025042.2	234 aa	DEFINITION R-spondin-4 isoform 1 precursor [Homo sapiens].<br>ACCESSION: NP_001025042 XP_297816<br>VERSION: NP_001025042.2 GI:83722284

Supplementary Table 2

Table S2 (Rspo)s and their genes

No	Gene/Locus	Gene ID	Gene Location	Protein (Gene product)
1	*RSPO1*	Gene ID: 284654	Chromosome: 1; 1p34.3<br>(38076821..38100595)	R-spondin 1
2	*RSPO2*	Gene ID: 340419	Chromosome: 8; 8q23.1<br>(108911544..109095913)	R-spondin 2
3	*RSPO3*	Gene ID: 84870	Chromosome: 6; 6q22.33<br>(127439844..127520626)	R-spondin 3
4	*RSPO4*	Gene ID: 343637	Chromosome: 20;20p13<br>(939095..982907)	R-spondin 4

Supplementary Table S3- (Rspo)s and their compositional analysis, charge distribution analysis, repetitive structures, and cysteine positions

Name	Mol. wt. (kdal)	Compositional analysis of amino acids	Charge Distribution Analysis	Repetitive Structures	cysteine positions
R-spondin-1	29.0 kdal	A : 19(7.2%); C++: 23(8.7%); D- : 5(1.9%); E : 18(6.8%); F : 5(1.9%)<br><br>G : 26(9.9%); H : 6(2.3%); I : 7(2.7%); K : 19(7.2%); L : 17(6.5%)<br><br>M : 4(1.5%); N : 9(3.4%); P : 15(5.7%); Q : 15(5.7%); R+ : 27(10.3%)<br><br>S : 21(8.0%); T : 10(3.8%); V : 11(4.2%); W : 3(1.1%); Y : 3(1.1%)	0+00000000 0000000000 0+00+0++0+ +000-00000 0+00-000-0 0000+000+0<br><br>0000-+0-0+ 0000000000 0000-0+00- 00+00+0+0-00-0000000 00+0+-0000<br><br>0+0+000000 -000000000 -0000000-0 0-00000000 ++000000++ 00--+0++00<br><br>00000-0000 0-0+-0++00 0++0000-00 ++++0000++ -000+000++ -0+-00000+<br><br>+++0000000 0000000000 000	A. SEPARATED, TANDEM, AND PERIODIC REPEATS: amino acid alphabet.<br><br>Repeat core block length: 4<br><br>Aligned matching blocks:<br><br>[129- 132] CPEG<br><br>[206- 209] CPEG<br><br>[212- 215] RRKG<br><br>[241- 244] RRKG<br><br>B. SEPARATED AND TANDEM REPEATS: 11-letter reduced alphabet.<br><br>(i= LVIF; += KR; -= ED; s= AG; o= ST; n= NQ; a= YW; p= P; h= H; m= M; c= C)<br><br>Repeat core block length: 8	6,40,44,47,53,56,79,94,97,102,105,111,125,129,142,148,159,166,190,199

R-spondin-2	28.3 kdal	A : 10(4.1%); C++: 24(9.9%); D : 9(3.7%); E : 15(6.2%); F : 13(5.3%)<br><br>G : 17(7.0%); H : 7(2.9%); I : 8(3.3%); K : 21(8.6%); L- : 13(5.3%)<br><br>M : 7(2.9%); N : 10(4.1%); P : 9(3.7%); Q : 9(3.7%); R+ : 27(11.1%)<br><br>S : 16(6.6%); T : 11(4.5%); V- : 7(2.9%); W : 4(1.6%); Y : 6(2.5%)	000+000000 000000-000 0000+0++0+ +000000000 +000000+-0 000+000+00<br><br>000++-00+0 00-0000000 00000+00-0 0+00+0+0-0 0-0000+-00 0+0+000000<br><br>+0+00--00- 00000--00- 00-00-0000 0-00000+00 +0000+000-0+0+000++0<br><br>0+-0000000 0-0++0+000 +00000++00 +0+-++0+++ +++00-+00-00000000-+<br><br>000	*A. SEPARATED, TANDEM, AND PERIODIC REPEATS: amino acid alphabet.*<br><br>Repeat core block length: 4<br><br>*B. SEPARATED AND TANDEM REPEATS: 11-letter reduced alphabet.*<br><br>(i= LVIF; += KR; -= ED; s= AG; o= ST; n= NQ; a= YW; p= P; h= H; m= M; c= C)<br><br>Repeat core block length: 8	15,21,46,52,55,74,78,93,96,101,104,110,113,124,128,141,145,156,163,187,196,203
R-spondin-3	30.9 kdal	A- : 7(2.6%); C++: 22(8.1%); D : 7(2.6%); E : 18(6.6%); F : 8(2.9%)<br><br>G : 21(7.7%); H : 7(2.6%); I : 12(4.4%); K+ : 33(12.1%); L- : 14(5.1%)<br><br>M : 5(1.8%); N : 15(5.5%); P : 13(4.8%); Q : 14(5.1%); R : 20(7.4%)<br><br>S : 22(8.1%); T : 14(5.1%); V	000+000000 000000-000 00000+0++0 ++00000000 000000000-0000000+0+<br><br>00000-+000 +000000000 0000000+00 -00+00+0+0 -0-0000+00 00+0+00000<br><br>000+00-000 -00-000000 -0000000-0 0-00000000 ++0+0000++ 00-0+0+-00<br><br>00000+0000 0000-0++00 00+++00+0-+0++0+-+++ ++00+0-0+- 000-0+00-0<br><br>0+-00-0+-0 +000++++00 -	A. SEPARATED, TANDEM, AND PERIODIC REPEATS: amino acid alphabet.<br><br>Repeat core block length: 4<br><br>Aligned matching blocks:<br><br>[93- 99] NK__CTKCK<br><br>[107- 115] NKNFCTKCK<br><br>B. SEPARATED AND TANDEM REPEATS: 11-letter reduced alphabet.<br><br>(i= LVIF; += KR; -= ED; s= AG; o= ST; n= NQ; a= YW; p= P; h= H; m= M; c= C)<br><br>Repeat core block length: 8	41,45,48,54,57,76,80,95,102,105,111,114,125,129,142,148,159,166,190,199

		: 11(4.0%); W : 3(1.1%); Y : 6(2.2%)	+0+000000 00		
R-spondin-4	26.2 kdal	A : 11(4.7%); C++: 23(9.8%); D : 7(3.0%); E : 14(6.0%); F : 6(2.6%)<br><br>G+ : 27(11.5%); H : 5(2.1%); I : 7(3.0%); K : 15(6.4%); L : 20(8.5%)<br><br>M : 2(0.9%); N : 6(2.6%); P : 16(6.8%); Q : 14(6.0%); R++: 27(11.5%)<br><br>S : 10(4.3%); T : 10(4.3%); V- : 7(3.0%); W : 3(1.3%); Y : 4(1.7%)	0+00000000 00000-0000 0++++00000 0000000000 00--000000 00+00000++<br><br>-00+000+00 0-00000000 +00-00+0++ 00000-0000 0-000+0++0 0000+0+000<br><br>0000000000 00+-000-0- 0000000000 0000+00000 000-0+0+-0 0+000--000<br><br>00000-0++0 000+0000-+ 0000++0++- ++0++-++0- ++0-0+0+00 0000	A. SEPARATED, TANDEM, AND PERIODIC REPEATS: amino acid alphabet.<br><br>Repeat core block length: 4<br><br>Aligned matching blocks:<br><br>[67- 76] GKCLHDCPPG<br><br>[116- 125] GKCLPTCPPG<br><br>[208- 211] RKDR<br><br>[214- 217] RKDR<br><br>B. SEPARATED AND TANDEM REPEATS: 11-letter reduced alphabet.<br><br>(i= LVIF; += KR; -= ED; s= AG; o= ST; n= NQ; a= YW; p= P; h= H; m= M; c= C)<br><br>Repeat core block length: 8	6,35,38,41,47,50,73,88,91,95,98,104,118,122,135,139,150,157,181,190

Supplementary Table 4

Table S4- Phosphorylation sites of (Rspo)s

R-spondin-1

Phosphorylation sites predicted: Ser: 7 Thr: 4 Tyr: 2

Serine predictions

Name	Pos	Context	Score	Pred
R_spondin_1	13	ALVLSWTHL	0.031	.
R_spondin_1	20	HLTISSRGI	0.925	*S*
R_spondin_1	21	LTISSRGIK	0.003	.
R_spondin_1	33	QRRISAEGS	0.998	*S*
R_spondin_1	37	SAEGSQACA	0.517	*S*
R_spondin_1	48	CELCSEVNG	0.047	.
R_spondin_1	57	CLKCSPKLF	0.277	.
R_spondin_1	78	VCLPSCPPG	0.010	.
R_spondin_1	107	EACFSHNFC	0.003	.
R_spondin_1	133	CPEGSSAAN	0.009	.
R_spondin_1	134	PEGSSAANG	0.029	.
R_spondin_1	143	TMECSSPAQ	0.010	.
R_spondin_1	144	MECSSPAQC	0.494	.
R_spondin_1	151	QCEMSEWSP	0.023	.
R_spondin_1	154	MSEWSPWGP	0.313	.
R_spondin_1	160	WGPCSKKQQ	0.537	*S*
R_spondin_1	172	FRRGSEERT	0.998	*S*
R_spondin_1	191	HAACSDTKE	0.143	.
R_spondin_1	232	ARKESKEAG	0.993	*S*
R_spondin_1	239	AGAGSRRRK	0.794	*S*
R_spondin_1	259	GPLTSAGPA	0.013	.

Threonine predictions

Name	Pos	Context	Score	Pred
R_spondin_1	15	VLSWTHLTI	0.004	.
R_spondin_1	18	WTHLTISSR	0.507	*T*
R_spondin_1	112	HNFCTKCKE	0.499	.
R_spondin_1	139	AANGTMECS	0.023	.
R_spondin_1	176	SEERTRRVL	0.926	*T*
R_spondin_1	193	ACSDTKETR	0.047	.
R_spondin_1	196	DTKETRRCT	0.888	*T*
R_spondin_1	200	TRRCTVRRV	0.990	*T*
R_spondin_1	253	QQQGTVGPL	0.128	.
R_spondin_1	258	VGPLTSAGP	0.421	.

Tyrosine predictions

Name	Pos	Context	Score	Pred
R_spondin_1	83	CPPGYFDAR	0.897	*Y*
R_spondin_1	119	KEGLYLHKG	0.923	*Y*
R_spondin_1	126	KGRCYPACP	0.019	.

R-spondin-2

Phosphorylation sites predicted:Ser: 4 Thr: 1 Tyr: 1

Serine predictions

Name	Pos	Context	Score	Pred
R_spondin_2	7	FRLFSFALI	0.005	.
R_spondin_2	19	CMDYSHCQG	0.005	.
R_spondin_2	29	RWRRSKRAS	0.988	*S*
R_spondin_2	33	SKRASYVSN	0.997	*S*
R_spondin_2	36	ASYVSNPIC	0.015	.
R_spondin_2	45	KGCLSCSKD	0.006	.
R_spondin_2	47	CLSCSKDNG	0.093	.
R_spondin_2	53	DNGCSRCQQ	0.152	.
R_spondin_2	77	ECLHSCPSG	0.064	.
R_spondin_2	80	HSCPSGYYG	0.047	.
R_spondin_2	103	ENCDSCFSK	0.040	.
R_spondin_2	106	DSCFSKDFC	0.007	.
R_spondin_2	151	VGHWSEWGT	0.030	.
R_spondin_2	157	WGTCSRNNR	0.003	.
R_spondin_2	193	TIAESRRCK	0.788	*S*
R_spondin_2	233	QEQHSVFLA	0.555	*S*

Threonine predictions

Name	Pos	Context	Score	Pred
R_spondin_2	111	KDFCTKCKV	0.344	.
R_spondin_2	138	PLEETMECV	0.171	.
R_spondin_2	155	SEWGTCSRN	0.106	.
R_spondin_2	162	RNNRTCGFK	0.007	.
R_spondin_2	171	WGLETRTRQ	0.076	.
R_spondin_2	173	LETRTRQIV	0.020	.
R_spondin_2	184	PVKDTILCP	0.097	.
R_spondin_2	189	ILCPTIAES	0.098	.
R_spondin_2	199	RCKMTMRHC	0.398	.
R_spondin_2	209	GGKRTPKAK	0.880	*T*
R_spondin_2	238	VFLATDRAN	0.213	.

Tyrosine predictions

Name	Pos	Context	Score	Pred
R_spondin_2	18	NCMDYSHCQ	0.095	.
R_spondin_2	34	KRASYVSNP	0.941	*Y*
R_spondin_2	71	GMRQYGECL	0.041	.
R_spondin_2	82	CPSGYYGHR	0.129	.
R_spondin_2	83	PSGYYGHRA	0.190	.
R_spondin_2	118	KVGFYLHRG	0.031	.

R-spondin-3

Phosphorylation sites predicted: Ser: 8 Thr: 5 Tyr: 1

Serine predictions

Name	Pos	Context	Score	Pred
R_spondin_3	7	LRLISWLFI	0.022	.
R_spondin_3	21	EYIGSQNAS	0.011	.
R_spondin_3	25	SQNASRGRR	0.350	.
R_spondin_3	38	HPNVSQGCQ	0.722	*S*
R_spondin_3	49	CATCSDYNG	0.056	.
R_spondin_3	56	NGCLSCKPR	0.147	.
R_spondin_3	78	GVCLSSCPS	0.034	.
R_spondin_3	79	VCLSSCPSG	0.019	.
R_spondin_3	82	SSCPSGYYG	0.120	.
R_spondin_3	116	TKCKSGFYL	0.010	.
R_spondin_3	144	MECVSIVHC	0.052	.
R_spondin_3	151	HCEVSEWNP	0.007	.
R_spondin_3	157	WNPWSPCTK	0.042	.
R_spondin_3	184	IQHPSAKGN	0.785	*S*
R_spondin_3	228	NKGESKEAI	0.994	*S*
R_spondin_3	235	AIPDSKSLE	0.238	.
R_spondin_3	237	PDSKSLESS	0.995	*S*
R_spondin_3	240	KSLESSKEI	0.997	*S*
R_spondin_3	241	SLESSKEIP	0.978	*S*
R_spondin_3	265	DKQKSVSVS	0.912	*S*
R_spondin_3	267	QKSVSVSTV	0.833	*S*
R_spondin_3	269	SVSVSTVH-	0.437	.

Threonine predictions

Name	Pos	Context	Score	Pred
R_spondin_3	47	GGCATCSDY	0.122	.
R_spondin_3	87	GYYGTRYPD	0.545	*T*
R_spondin_3	96	INKCTKCKA	0.286	.
R_spondin_3	104	ADCDTCFNK	0.035	.
R_spondin_3	112	KNFCTKCKS	0.204	.
R_spondin_3	139	ANNHTMECV	0.006	.
R_spondin_3	160	WSPCTKKGK	0.822	*T*
R_spondin_3	165	KKGKTCGFK	0.017	.
R_spondin_3	172	FKRGTETRV	0.727	*T*
R_spondin_3	174	RGTETRVRE	0.844	*T*
R_spondin_3	193	LCPPTNETR	0.008	.
R_spondin_3	196	PTNETRKCT	0.705	*T*
R_spondin_3	200	TRKCTVQRK	0.107	.
R_spondin_3	270	VSVSTVH--	0.110	.

Tyrosine predictions

Name	Pos	Context	Score	Pred
R_spondin_3	18	NFMEYIGSQ	0.064	.
R_spondin_3	51	TCSDYNGCL	0.108	.
R_spondin_3	84	CPSGYYGTR	0.131	.
R_spondin_3	85	PSGYYGTRY	0.199	.
R_spondin_3	89	YGTRYPDIN	0.883	*Y*
R_spondin_3	119	KSGFYLHLG	0.228	.

R-spondin-4

Phosphorylation sites predicted: Ser: 2 Thr: 0 Tyr: 0

Serine predictions

Name	Pos	Context	Score	Pred
R_spondin_4	42	CIICSEENG	0.190	.
R_spondin_4	48	ENGCSTCQQ	0.088	.
R_spondin_4	97	ATCESCFSQ	0.041	.
R_spondin_4	100	ESCFSQDFC	0.007	.
R_spondin_4	148	WGGWSPCTH	0.013	.
R_spondin_4	159	KTCGSAWGL	0.021	.
R_spondin_4	165	WGLESRVRE	0.091	.
R_spondin_4	185	CQVLSESRK	0.851	*S*
R_spondin_4	187	VLSESRKCP	0.945	*S*
R_spondin_4	201	PGERSPGQK	0.403	.

Threonine predictions

Name	Pos	Context	Score	Pred
R_spondin_4	29	KQVGTGLGG	0.039	.
R_spondin_4	36	GGNCTGCII	0.013	.
R_spondin_4	49	NGCSTCQQR	0.032	.
R_spondin_4	94	KCGATCESC	0.021	.
R_spondin_4	121	KCLPTCPPG	0.013	.
R_spondin_4	126	CPPGTLAHQ	0.040	.
R_spondin_4	132	AHQNTRECQ	0.026	.
R_spondin_4	151	WSPCTHNGK	0.117	.
R_spondin_4	156	HNGKTCGSA	0.076	.
R_spondin_4	180	EEAATCQVL	0.093	.

Tyrosine predictions

Name	Pos	Context	Score	Pred
R_spondin_4	66	GIRQYGKCL	0.007	.
R_spondin_4	77	CPPGYFGIR	0.457	.
R_spondin_4	112	KRQFYLYKG	0.096	.
R_spondin_4	114	QFYLYKGKC	0.026	.

Supplementary Table 5

Table S5- N-glycosylation sites of (Rspo)s

R-spondin-1

(Threshold=0.5)

SeqName	Position	Potential	Jury agreement	N-Glyc result
R-spondin-1	137 NGTM	0.6591	(8/9)	+

R-spondin-2

(Threshold=0.5)

SeqName	Position	Potential	Jury agreement	N-Glyc result
R-spondin-2	160 NRTC	0.6008	(6/9)	+

R-spondin-3

(Threshold=0.5)

SeqName	Position	Potential	Jury agreement	N-Glyc result
R-spondin-3	23 NASR	0.5513	(5/9)	+
R-spondin-3	36 NVSQ	0.7283	(9/9)	++
R-spondin-3	137 NHTM	0.5899	(6/9)	+
R-spondin-3	194 NETR	0.5246	(7/9)	+

R-spondin-4

(Threshold=0.5)

SeqName	Position	Potential	Jury agreement	N-Glyc result
R-spondin-4	34 NCTG	0.6842	(9/9)	++

Supplementary Table 6

Table S6- O-glycosylation sites of (Rspo)s

R-spondin-1

Name	S/T	Pos	G-score	I-score	Y/N	Comment
R_spondin_1	S	13	0.123	0.044	.	-
R_spondin_1	T	15	0.173	0.052	.	-
R_spondin_1	T	18	0.208	0.055	.	-
R_spondin_1	S	20	0.168	0.055	.	-
R_spondin_1	S	21	0.190	0.078	.	-
R_spondin_1	S	33	0.241	0.053	.	-
R_spondin_1	S	37	0.190	0.038	.	-
R_spondin_1	S	48	0.159	0.062	.	-
R_spondin_1	S	57	0.093	0.067	.	-
R_spondin_1	S	78	0.178	0.253	.	-
R_spondin_1	S	107	0.076	0.057	.	-
R_spondin_1	T	112	0.150	0.052	.	-
R_spondin_1	S	133	0.296	0.057	.	-
R_spondin_1	S	134	0.316	0.133	.	-
R_spondin_1	T	139	0.509	0.059	T	-
R_spondin_1	S	143	0.465	0.039	.	-
R_spondin_1	S	144	0.425	0.026	.	-
R_spondin_1	S	151	0.296	0.048	.	-
R_spondin_1	S	154	0.277	0.035	.	-
R_spondin_1	S	160	0.243	0.032	.	-
R_spondin_1	S	172	0.221	0.026	.	-
R_spondin_1	T	176	0.270	0.058	.	-
R_spondin_1	S	191	0.298	0.042	.	-
R_spondin_1	T	193	0.443	0.044	.	-
R_spondin_1	T	196	0.438	0.076	.	-
R_spondin_1	T	200	0.365	0.031	.	-
R_spondin_1	S	232	0.199	0.043	.	-
R_spondin_1	S	239	0.230	0.030	.	-
R_spondin_1	T	253	0.491	0.288	.	-
R_spondin_1	T	258	0.551	0.078	T	-
R_spondin_1	S	259	0.484	0.383	.	-

R-spondin-2

Name	S/T	Pos	G-score	I-score	Y/N	Comment
R_spondin_2	S	7	0.115	0.054	.	-
R_spondin_2	S	19	0.092	0.041	.	-
R_spondin_2	S	29	0.142	0.031	.	-
R_spondin_2	S	33	0.168	0.034	.	-
R_spondin_2	S	36	0.155	0.080	.	-
R_spondin_2	S	45	0.136	0.053	.	-
R_spondin_2	S	47	0.139	0.059	.	-
R_spondin_2	S	53	0.102	0.029	.	-
R_spondin_2	S	77	0.153	0.053	.	-
R_spondin_2	S	80	0.164	0.037	.	-
R_spondin_2	S	103	0.114	0.021	.	-
R_spondin_2	S	106	0.092	0.061	.	-
R_spondin_2	T	111	0.139	0.053	.	-
R_spondin_2	T	138	0.310	0.042	.	-
R_spondin_2	S	151	0.179	0.033	.	-
R_spondin_2	T	155	0.234	0.043	.	-
R_spondin_2	S	157	0.168	0.032	.	-
R_spondin_2	T	162	0.268	0.035	.	-
R_spondin_2	T	171	0.249	0.033	.	-
R_spondin_2	T	173	0.246	0.063	.	-
R_spondin_2	T	184	0.328	0.062	.	-
R_spondin_2	T	189	0.304	0.178	.	-
R_spondin_2	S	193	0.190	0.055	.	-
R_spondin_2	T	199	0.377	0.055	.	-
R_spondin_2	T	209	0.241	0.042	.	-
R_spondin_2	S	233	0.167	0.037	.	-
R_spondin_2	T	238	0.382	0.065	.	-

R-spondin-3

Name	S/T	Pos	G-score	I-score	Y/N	Comment
R_spondin_3	S	7	0.113	0.054	.	-
R_spondin_3	S	21	0.110	0.054	.	-
R_spondin_3	S	25	0.151	0.042	.	-
R_spondin_3	S	38	0.197	0.116	.	-
R_spondin_3	T	47	0.238	0.030	.	-
R_spondin_3	S	49	0.166	0.034	.	-
R_spondin_3	S	56	0.105	0.056	.	-
R_spondin_3	S	78	0.161	0.103	.	-
R_spondin_3	S	79	0.158	0.034	.	-
R_spondin_3	S	82	0.164	0.034	.	-
R_spondin_3	T	87	0.240	0.057	.	-
R_spondin_3	T	96	0.208	0.030	.	-
R_spondin_3	T	104	0.170	0.020	.	-
R_spondin_3	T	112	0.116	0.051	.	-
R_spondin_3	S	116	0.082	0.032	.	-
R_spondin_3	T	139	0.206	0.045	.	-
R_spondin_3	S	144	0.225	0.036	.	-
R_spondin_3	S	151	0.198	0.034	.	-
R_spondin_3	S	157	0.241	0.050	.	-
R_spondin_3	T	160	0.363	0.048	.	-
R_spondin_3	T	165	0.391	0.055	.	-
R_spondin_3	T	172	0.378	0.034	.	-
R_spondin_3	T	174	0.288	0.032	.	-
R_spondin_3	S	184	0.276	0.154	.	-
R_spondin_3	T	193	0.342	0.066	.	-
R_spondin_3	T	196	0.341	0.073	.	-
R_spondin_3	T	200	0.278	0.025	.	-
R_spondin_3	S	228	0.298	0.044	.	-
R_spondin_3	S	235	0.345	0.060	.	-
R_spondin_3	S	237	0.344	0.021	.	-
R_spondin_3	S	240	0.302	0.043	.	-
R_spondin_3	S	241	0.309	0.058	.	-
R_spondin_3	S	265	0.291	0.062	.	-
R_spondin_3	S	267	0.343	0.058	.	-
R_spondin_3	S	269	0.370	0.085	.	-
R_spondin_3	T	270	0.478	0.029	.	-

R-spondin-4

Name	S/T	Pos	G-score	I-score	Y/N	Comment
R_spondin_4	T	29	0.198	0.101	.	-
R_spondin_4	T	36	0.221	0.037	.	-
R_spondin_4	S	42	0.142	0.073	.	-
R_spondin_4	S	48	0.108	0.023	.	-
R_spondin_4	T	49	0.159	0.028	.	-
R_spondin_4	T	94	0.175	0.023	.	-
R_spondin_4	S	97	0.107	0.021	.	-
R_spondin_4	S	100	0.089	0.055	.	-
R_spondin_4	T	121	0.227	0.456	.	-
R_spondin_4	T	126	0.288	0.057	.	-
R_spondin_4	T	132	0.389	0.055	.	-
R_spondin_4	S	148	0.236	0.036	.	-
R_spondin_4	T	151	0.334	0.051	.	-
R_spondin_4	T	156	0.377	0.031	.	-
R_spondin_4	S	159	0.260	0.026	.	-
R_spondin_4	S	165	0.297	0.032	.	-
R_spondin_4	T	180	0.427	0.088	.	-
R_spondin_4	S	185	0.325	0.068	.	-
R_spondin_4	S	187	0.374	0.059	.	-
R_spondin_4	S	201	0.326	0.031	.	-
