# Supplementary material for: Computational Biophysical, Biochemical, and Evolutionary Signature of Human R-Spondin Family Proteins, the Member of Canonical Wnt/β-Catenin Signaling Pathway
Source: Biomed Res Int. 2014 Sep 8;2014:974316. doi: 10.1155/2014/974316 (PMC4172882; doi:10.1155/2014/974316)
Supplement: Supplementary file 2 [file 974316.f2.pdf]

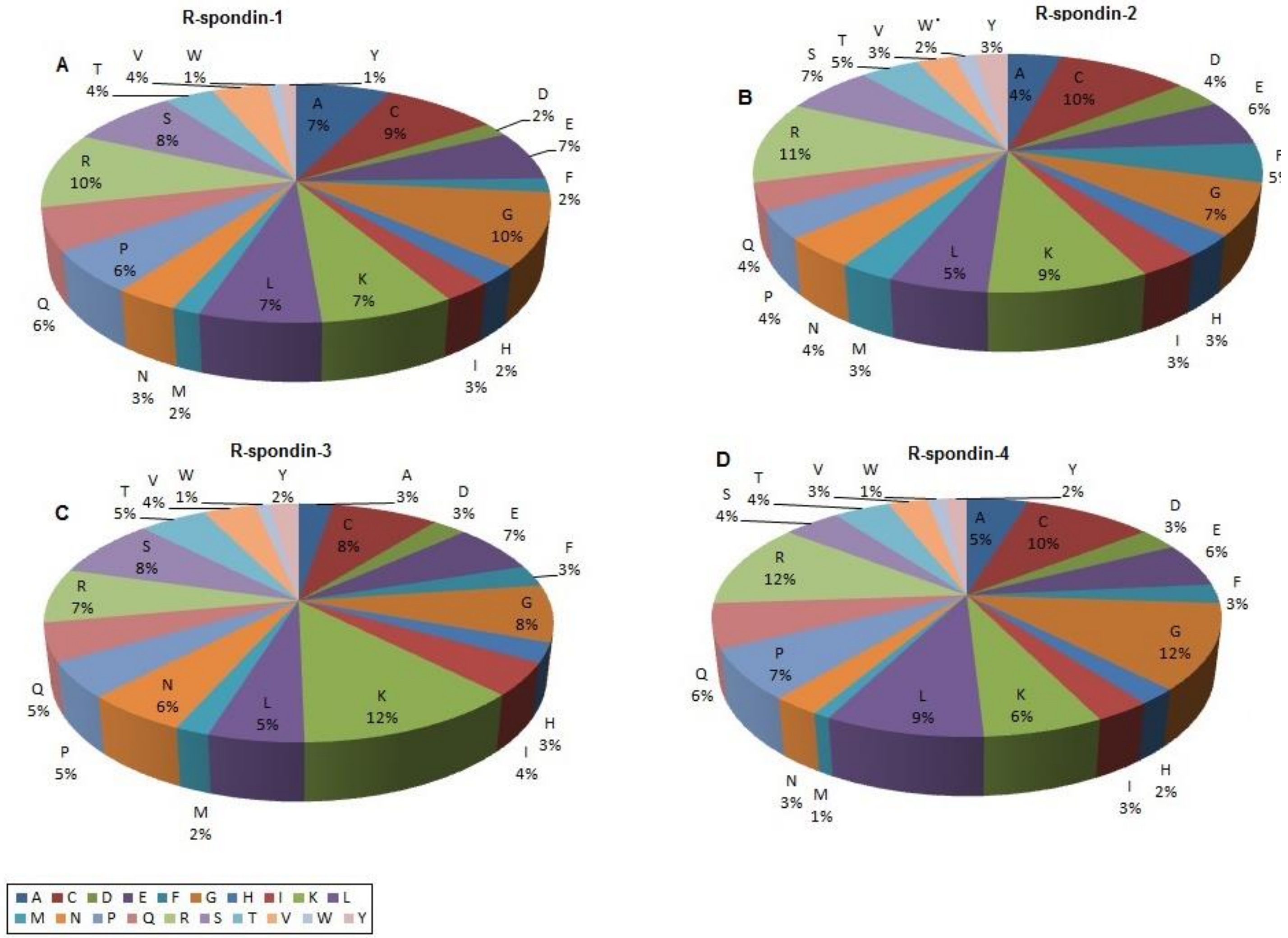

Supplementary Figure- S1- Compositional analysis of (Rspo)s

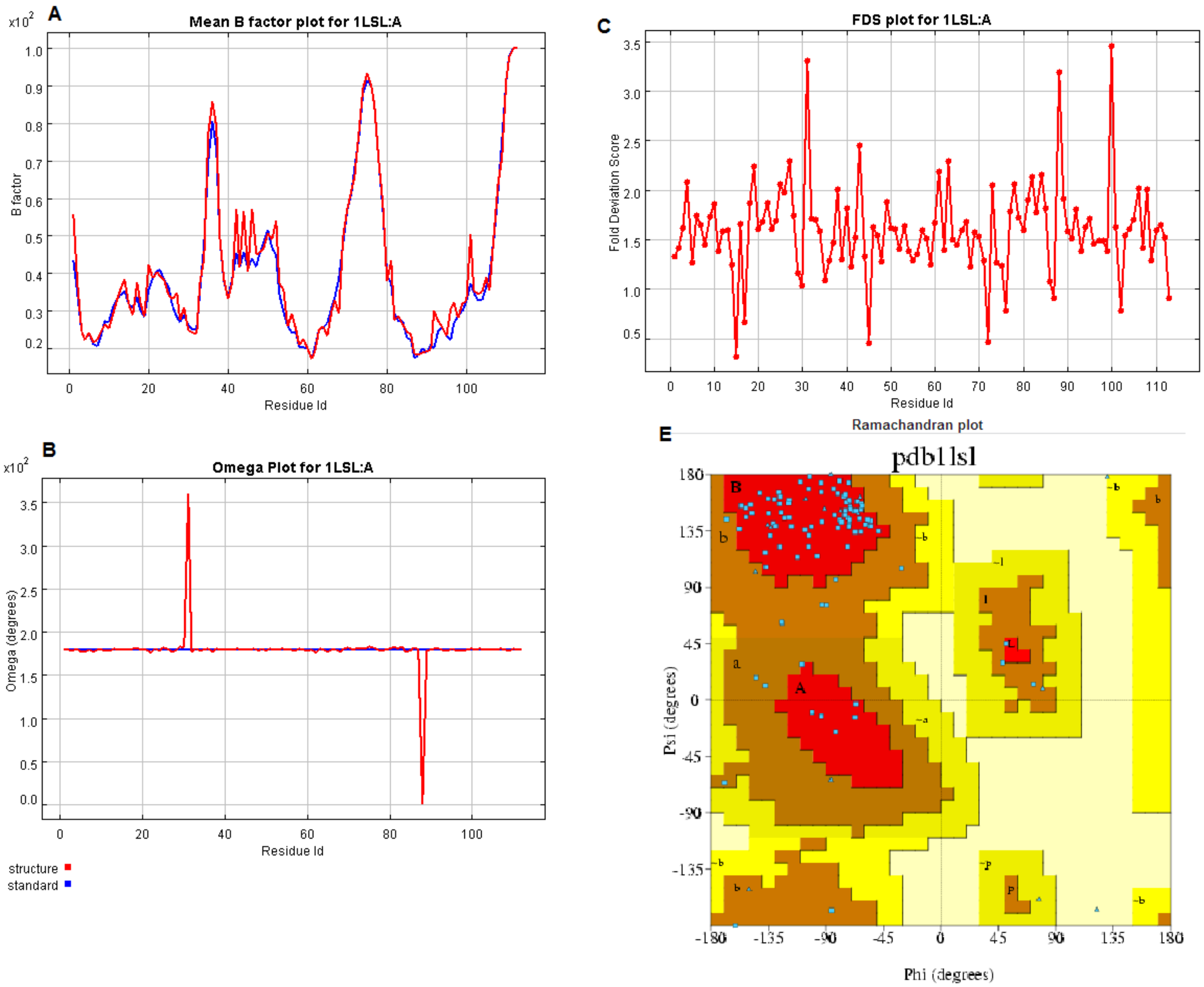

Supplementary Figure- S2: Computational analysis of the geometry of the thrombospondin-1 domain type 1 (A) B factor plot, (B)Omega plot (C) FDS (fold deviation score) plot (D) Ramachandran plot

```
CLUSTAL O(1.2.0) multiple sequence alignment


R-spondin-4      MRAPLCLLLL-VAHAVDMLA------LNRRKKQVGTGLGGNCTGCIICSEENGCSTCQQR
R-spondin-2      MQFRLFSFALIIILNCMDYSHCQGNR-WRRSKR-ASYVSNPICKGCLSCSKDNGCSRCQQK
R-spondin-1      MRLGLC-VVALVLSWTHLTISSRGIKGKRQRRISAEGSQACAKGCELCSEVNGCLKCSPK
R-spondin-3      MHLRLISWLFIILNFMEYIGSQNASRGRRQRRMHPNVSQGCQGGCATCSDYNGCLSCKPR
                 *:  *      :    .          .* ::           **  **. ***  *. :

R-spondin-4      LFLFIRREGIRQYGKCLHDCPPGYFGIRGQEVNRCKKCGA-TCESCFSQDFCIRCKRQFY
R-spondin-2      LFFFLRREGMRQYGECLHSCPSGYYGHRAPDMNRCARCRIENCDSCFSKDFCTKCKVGFY
R-spondin-1      LFILLERNDIRQVGVCLPSCPPGYFDARNPDMNKCIKCKIEHCEACFSHNFCTKCKEGLY
R-spondin-3      LFFALERIGMKQIGVCLSSCPSGYYGTRYPDINKCTKCKA-DCDTCFNKNFCTKCKSGFY
                 **: :.*  ::* * ** .** **:  *  ::*:* :*    *::**.::** :**  :*

R-spondin-4      LYKGKCLPTCPPGTLAHQNTRECQG--ECELGPWGGWSPCTHNGKTCGSAWGLESRVREA
R-spondin-2      LHRGRCFDECPDGFAPLEETMECVE--GCEVGHWSEWGTCSRNNRTCGFKWGLETRTRQI
R-spondin-1      LHKGRCYPACPEGSSAANGTMECSSPAQCEMSEWSPWGPCSKKQQLCGFRRGSEERTRRV
R-spondin-3      LHLGKCLDNCPEGLEANNHTMECVSIVHCEVSEWNPWSPCTKKGKTCGFKRGTETRVREI
                 *: *:*   ** *    : * **     **:. *  *. *::: : **   * * *.*.

R-spondin-4      GRAGHEEAATCQVLSESRKCPIQR-PCPGERSPGQKKGRKDRRPRKDRKLDRRLDVR---
R-spondin-2      VKKPVKDTILCPTIAESRRCKMTMRHCPGGKRT--PKAKEKRNKKKKRKLIERAQEQHSV
R-spondin-1      LHAPVGDHAACSDTKETRRCTVRRVPCPEGQKR--RKGGQGRRENANRNLARKESKE---
R-spondin-3      IQHPSAKGNLCPPTNETRKCTVQRKKCQKGERG--KKGRERKRKKPNKGESKEAIPDSKS
                  :    .   *    *:*:* :    *   .     *. : :. . .:   ..

R-spondin-4      --------PRQPGLQP----------------------
R-spondin-2      FLA-----TDRANQ------------------------
R-spondin-1      --AGAGS-RRRKGQQQQQ------QQGTVGPLTSAGPA
R-spondin-3      LESSKEIPEQRENKQQQKKRKVQDKQKSVSVSTVH---
                           .
```

Supplementary Figure S3- Sequence alignment of (Rspo)s using Clustal Omega

Input:

- **RSPO1** R-spondin homolog (Xenopus laevis); Activator of the beta-catenin signaling cascade, leading to TCF-dependent gene activation. Acts both in the canonical Wnt/beta-catenin-dependent pathway, possibly via a direct interaction with Wnt proteins, and in a Wnt-independent beta catenin pathway through a receptor signaling pathway that may not use frizzled/LRP receptors. Acts as a ligand for frizzled FZD8 and LRP6. May negatively regulate the TGF-beta pathway. Has a essential roles in ovary determination (263 aa) *(Homo sapiens)*
- **RSPO2** R-spondin 2 homolog (Xenopus laevis); Activator of the beta-catenin signaling cascade, leading to TCF-dependent gene activation. Acts both in the canonical Wnt/beta-catenin-dependent pathway, possibly via a direct interaction with Wnt proteins, and in a Wnt-independent beta catenin pathway through a receptor signaling pathway that may not use frizzled/LRP receptors. Probably also acts as a ligand for frizzled and LRP receptors (By similarity) (243 aa) *(Homo sapiens)*
- **RSPO3** R-spondin 3 homolog (Xenopus laevis); Activator of the beta-catenin signaling cascade, leading to TCF-dependent gene activation. Acts both in the canonical Wnt/beta-catenin-dependent pathway, possibly via a direct interaction with Wnt proteins, and in a Wnt-independent beta catenin pathway through a receptor signaling pathway that may not use frizzled/LRP receptors. Acts as a ligand for frizzled FZD8 and LRP6. May negatively regulate the TGF-beta pathway (By similarity) (272 aa) *(Homo sapiens)*
- **RSPO4** R-spondin family, member 4; Activator of the beta-catenin signaling cascade, leading to TCF-dependent gene activation. Acts both in the canonical Wnt/beta-catenin-dependent pathway, possibly via a direct interaction with Wnt proteins, and in a Wnt-independent beta catenin pathway through a receptor signaling pathway that may not use frizzled/LRP receptors (By similarity) (234 aa) *(Homo sapiens)*

Supplementary Figure-S4-Input file for protein –protein interaction analysis of (Rspo)s
